# High accuracy machine learning identification of fentanyl-relevant molecular compound classification via constituent functional group analysis

**DOI:** 10.1038/s41598-020-70471-7

**Published:** 2020-08-11

**Authors:** Mengyu Xu, Chun-Hung Wang, Anthony C. Terracciano, Artem E. Masunov, Subith S. Vasu

**Affiliations:** 1 Statistics and Data Science, University of Central Florida, 4000 Central Florida Blvd, Orlando, FL 32816 USA; 2 NanoScience Technology Center, University of Central Florida, 12424 Research Parkway, Orlando, FL 32826 USA; 3 Mechanical and Aerospace Engineering, University of Central Florida, 4000 Central Florida Blvd, Orlando, FL 32816 USA; 4 Center for Advanced Turbomachinery and Energy Research, University of Central Florida, 4000 Central Florida Blvd, Orlando, FL 32816 USA; 5 School of Modeling, Simulation, and Training, University of Central Florida, 3100 Technology Parkway, Orlando, FL 32816 USA; 6 Department of Chemistry, University of Central Florida, 4111 Libra Dr., Orlando, FL 32816 USA; 7 South Ural State University, Lenin pr. 76, Chelyabinsk, 454080 Russia; 8 National Research Nuclear University MEPhI, Kashirskoye shosse 31, Moscow, 115409 Russia

**Keywords:** Cheminformatics, Chemical safety

## Abstract

Fentanyl is an anesthetic with a high bioavailability and is the leading cause of drug overdose death in the U.S. Fentanyl and its derivatives have a low lethal dose and street drugs which contain such compounds may lead to death of the user and simultaneously pose hazards for first responders. Rapid identification methods of both known and emerging opioid fentanyl substances is crucial. In this effort, machine learning (ML) is applied in a systematic manner to identify fentanyl-related functional groups in such compounds based on their observed spectral properties. In our study, accurate infrared (IR) spectra of common organic molecules which contain functional groups that are constituents of fentanyl is determined by investigating the structure–property relationship. The average accuracy rate of correctly identifying the functional groups of interest is 92.5% on our testing data. All the IR spectra of 632 organic molecules are from National Institute of Standards and Technology (NIST) database as the training set and are assessed. Results from this work will provide Artificial Intelligence (AI) based tools and algorithms increased confidence, which serves as a basis to detect fentanyl and its derivatives.

## Introduction

Fentanyl (*N*-phenyl-*N*-[1-(2-phenylethyl)piperidin-4-yl]propionanilide)^1^ is a synthetic opioid with extensive medical applications in acute analgesia. Fentanyl is an agonist for the μ-opioid receptor with higher potency than morphine, exhibiting an effective dose (ED_95_) of 0.45–0.60 μg/kg in dilation and curettage owe able in part to its 92% bioavailability^2,3^. Once in the human body, metabolizing fentanyl begins with N-oxidative dealkylation and is eventually excreted within the urine^4,5^. Fentanyl can be administered via inhalation, ingestion, oral exposure, injection, or transdermal means^6^. Fentanyls which are primarily powdered crystalline substances, typically white, obtained outside of a pharmaceutical setting may exist as a white powder form mixed with heroin and cutting agents, or pressed into counterfeit opioid prescription pills^7^. Fentanyl may even be weaponized and formed into an aerosol^8^. Such widespread use of fentanyl as either a primary compound chemical weapon or adulterating substance within other drugs poses a significant threat for officers the public at large, officers responding to incidents, and others given its fentanyls ample means of exposure^9^.

A comprehensive review of fentanyl and its analogs has been done in by Armenian et al.^10^. Carfentanil^11^, sufentanil^12^, alfentanil^13^, and remifentanil^14^ are fentanyl analogs with more potency than fentanyl for medicinal use. These and other fentanyls all exhibit some common key functional groups.

**Figure 1:**
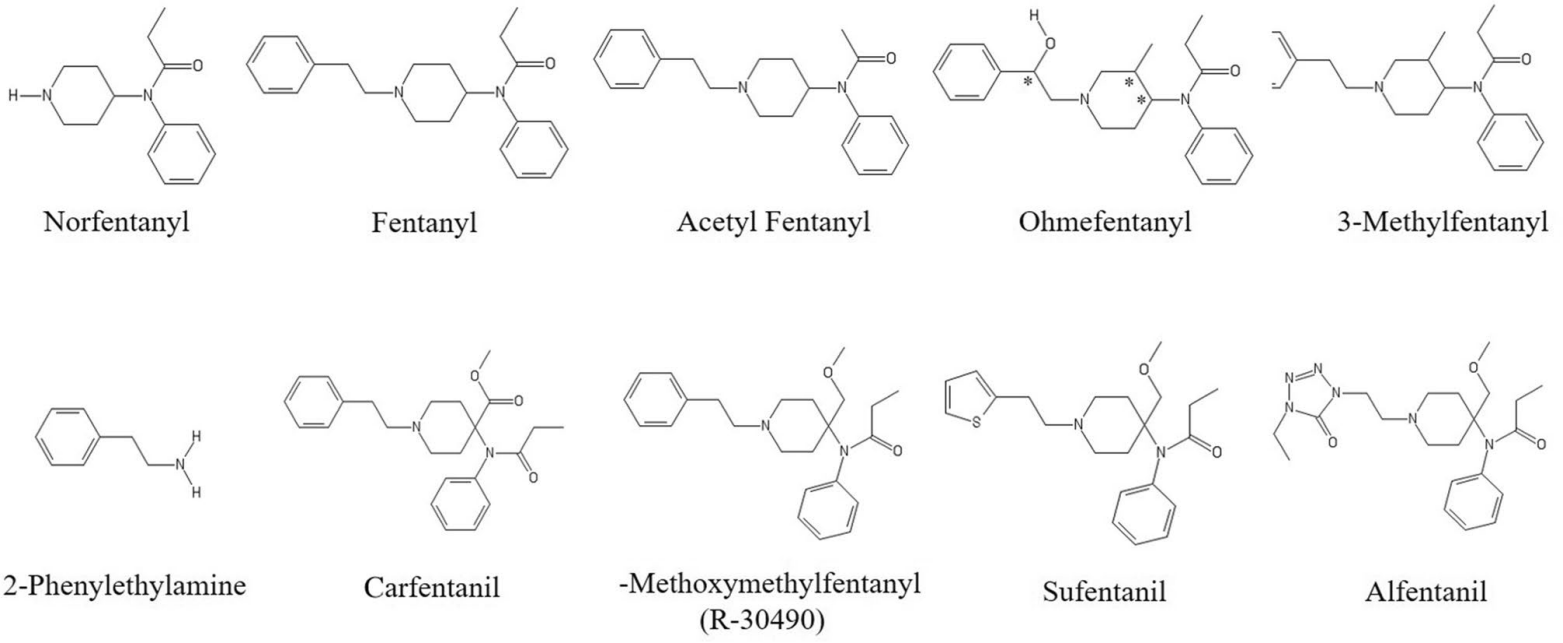
Fentanyl and selected analogues. * denotes chiral center of ohmefentanyl, which is a stereoisomer. Images generated using ChemDraw tool (PerkinElmer).

**Figure 2:**
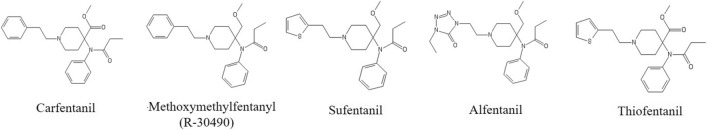
Carfentanil and selected analogues. Images generated using ChemDraw tool (PerkinElmer).

One promising method of identifying fentanyl and its analogues is through the fingerprinting of their unique infrared (IR) spectral properties (reflectivity, absorptivity, or transmissivity) as this technique can reveal characteristics of the numerous intramolecular bonds^15^. However, identifying fentanyls in practice is difficult. Clandestine production often exhibits variations in products between batches and as previously stated there are numerous formulations of fentanyl and carfentanil. As can be seen in Figs. 1 and 2, fentanyl, carfentanil, and their analogues exhibit varying functional groups and simultaneously may have conformers for the same composition which are denoted by a * at a particular bond. These structural variations can result in significantly different infrared spectral properties. Despite these differences, there are structural similarities enabling the compound to bind to receptors within the body and perform a similar function.

In analytical chemistry, IR spectroscopy tables^16^ had been widely used to identify compounds based on the correlations between IR absorption and common types of molecular bonds and functional groups. In this work, we aimed to answer the following question: can we identify constituent functional groups of fentanyl and its analogues from the IR absorption data?

**Figure 3:**
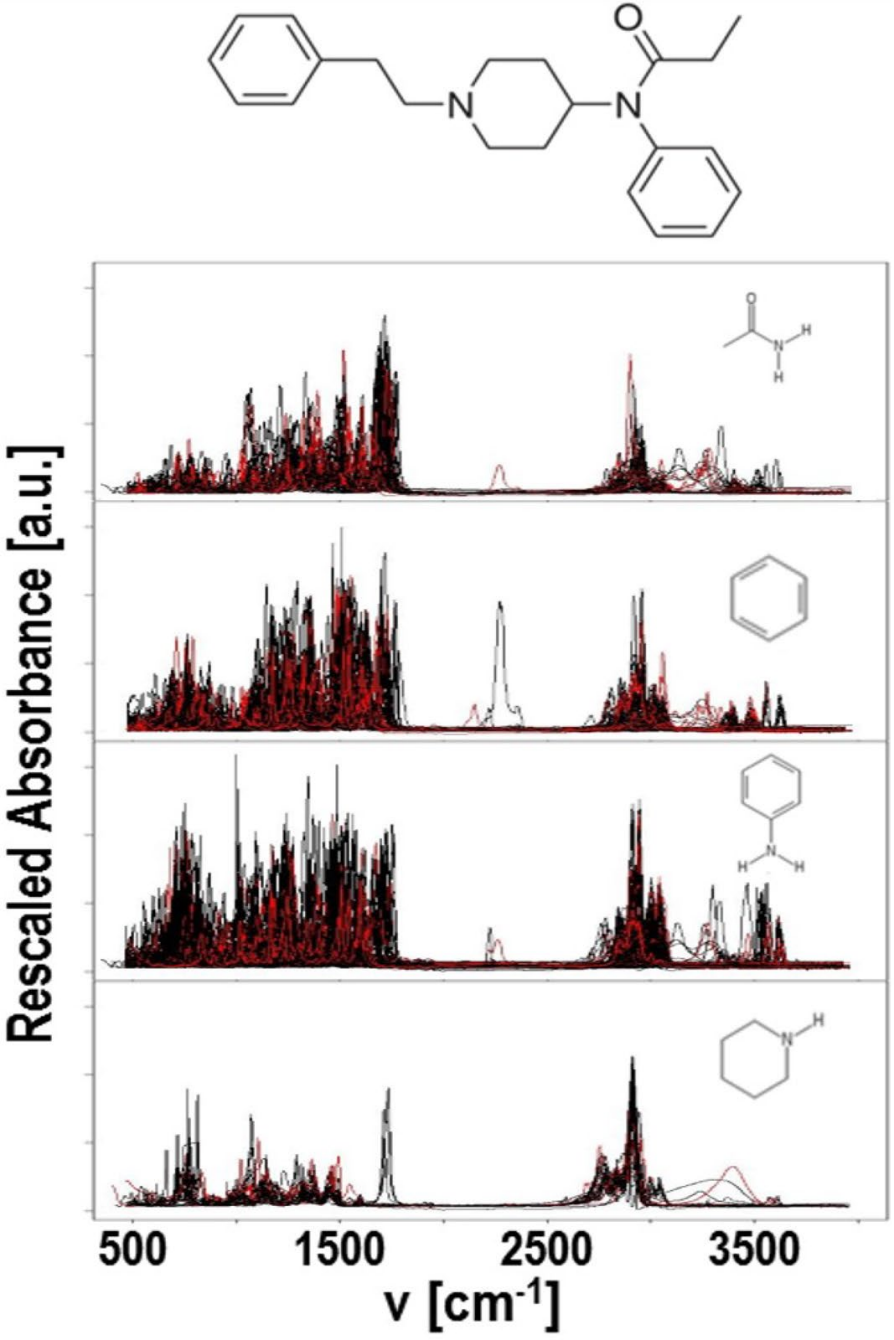
IR spectral curves of compounds with identified functional groups within fentanyl. Black: compounds in the training set; red: compounds in the test set. Image generated using ChemDraw tool (PerkinElmer).

Here we use a database containing 632 molecules, 591 of which have at least one of the functional groups found in the parent compound of fentanyl. The rescaled IR absorption spectra of these molecules are shown in Fig. 3. From these data we construct machine learning algorithms to identify molecules which are compromised from at least one of these functional groups.

A main challenge of learning from the spectral data is that the number of variables is the number of wavenumbers where absorptive data is obtained. The analysis suffers from a curse of dimensionality. There has been a growing body of literature in chemometrics that study the identification of compounds from their spectral properties via data-driven algorithms. In most of the work, spectral properties measured at each wavenumber is treated as a predictor, and widely-used classification or clustering machine learning methods has been applied for analysis directly or after dimension reduction with methods such as principal component analysis, see Refs.^17–24^ among others. In this work, we treat the underlying IR spectra as smooth functions and apply functional principal component analysis by approximating the data with a basis of a few orthogonal smoothed eigenfunction and then perform classification via functional generalized linear models. Comparing to the high-dimensional multivariate classification, the functional analysis is more interpretable. It associates the functional groups with features in the IR spectra in terms of the functional components along the whole range such as peaks and troughs. Results from this work will provide Artificial Intelligence (AI) based tools and algorithms increased confidence, which serves as a basis to detect fentanyl and its derivatives.

## Methodology

Let $\alpha_{1} ,\;\alpha_{2} , \ldots ,\alpha_{{\text{p}}}$ be the wavenumbers at which absorbance are recorded, $\nu_{{\text{i}}} (\alpha )$ be the underlying absorbance at any wavenumber ${\upalpha }$ for the ith molecule and $\nu_{{\text{i,j}}} = \nu_{{\text{i}}} (\alpha_{{\text{j}}} ) + \epsilon_{{\text{i,j}}} ,\;{\text{i}} = 1,\;2, \ldots ,{\text{n}},\;{\text{j}} = 1,\;2, \ldots ,{\text{p}}$ be the observed absorbance. The goal of this study is to distinguish if a given molecule contains a certain functional group such as amide. Let ${\text{y}}_{{\text{i}}} = 1({\text{molecule i contains the functional group}}),{ }\;{\text{i}} = 1,2, \ldots ,{\text{n}}$ be the binary observations. We establish a classification model with $\{ {\upnu }_{{{\text{i}},{\text{j}}}} \}_{{1 \le {\text{i}} \le {\text{n}},1 \le {\text{j}} \le {\text{p}}}}$ as the predictor and $\{ {\text{y}}_{{\text{i}}} \}_{{1 \le i \le n\user2{ }}}$ as the response, via a functional logistic regression with functional principal component basis.

### Dataset

The data set for both training and testing was obtained from the public database of National Institute of Standards and Technology (https://www.nist.gov/), which provides spectral information of the selected 632 compound molecules considered in this work., Such compounds can be categorized into one of the following eight groups: (i) amide only, (ii) aniline only, (iii) benzene only, (iv) piperidine only, (v) amide and aniline simultaneously, (vi) amide and benzene simultaneously (vii) distinct aniline and benzene simultaneously, and (viii) none of the above constituent functional groups. The group information of the data is listed in Table 1 with the names of the compounds listed in the Supplementary Appendix A.

**Table 1:** Number of molecules with the functional groups.

	Amide only	Aniline only	Benzene only	Piperidine only	Amide and aniline	Amide and Benzene	Aniline and Benzene	Benzene and Piperidine	None	Total
Training set	30	139	217	18	24	22	15	8	33	506
Testing set	7	35	54	5	6	5	4	2	8	126
Total	37	174	271	23	30	27	19	10	41	632

For each molecule, the discretization of the IR spectra is recorded with unaligned maximum wavenumbers ranges of 243 cm^−1^ to 4,000.7 cm^−1^ (7.28–119.94 THz), with the majority of the wavenumbers ranging from 500 to 4,000.7 cm^−1^ (14.99–119.94 THz). Preprocessing of the discrete raw data consists of rescaling each absorbance function such that it has unit standard deviation and converting it to regularly spaced measurement via interpolation at the common wavenumbers 500–4,000 cm^−1^ (14.99–119.92 THz) with step size 4 cm^−1^ (0.12 THz), yielding a total of $p = 876$ discrete points. For compounds with a slope in the IR spectra, we apply a linear baseline correction to remove the linear trend between the lowest point and the right end in the IR spectra. No subsequent smoothing of the data is performed in preprocessing. It is assumed that spectra obtained from the NIST database are close enough to standard temperature and pressure to neglect broadening and thermal effects. The preprocessed dataset is decomposed into distinct training and testing sets, consisting of 506 and 126 molecules, respectively, as shown in Table 1.

### Functional principal component analysis (functional PCA)

Denote $\overline{\nu }(\alpha ) = {\text{n}}^{ - 1} \nu_{{\text{i}}} (\alpha )$ as the mean function and $\sigma ({\text{s}},\;{\text{t}}) = ({\text{n}} - 1)^{ - 1} \sum\nolimits_{{{\text{i}} = 1}}^{{\text{n}}} {[{\upnu }_{{\text{i}}} ({\text{s}}) - \overline{\nu }({\text{s}})]} [{\upnu }_{{\text{i}}} ({\text{t}}) - \overline{\nu }({\text{t}})]$ as the covariance function of the sample. Functional PCA decomposes the underlying absorbance functional $\nu_{{\text{i}}} (\alpha )$ as.

1 $$ \nu (\alpha ) = \overline{\nu }(\alpha ) + \mathop \sum \limits_{{{\text{k}} = 1}}^{\infty } f_{{{\text{ik}}}} \phi_{{\text{k}}} (\alpha ), \quad {\text{i}} = 1,\;2, \ldots ,{\text{n}}, $$

where $\phi_{{\text{k}}} (\alpha ),\; {\text{k}} = 1,2, \ldots ,{\text{K}}$ are the functional principal components (PCs), and ${\text{f}}_{{{\text{ik}}}}$ is the score of molecule i for principal component k. Cutting off at a finite integer K and estimate $\nu_{{\text{i}}} (\alpha )$ according to

2 $$ \hat{\nu }_{{\text{i}}} (\alpha ) = \overline{\nu }(\alpha ) + \mathop \sum \limits_{{{\text{k}} = 1}}^{{\text{K}}} {\text{f}}_{{{\text{ik}}}} \phi_{{\text{k}}} (\alpha ),\quad {\text{ i}} = 1,\;2, \ldots ,{\text{n}}. $$

The functional PCs are the eigenfunctions of the covariance function, $\sigma ({\text{s}},\;{\text{t}})$, where $\phi_{{\text{k}}} (\alpha )$ corresponds to the kth largest eigenvalue $\lambda_{{\text{k}}}$. The corresponding PC scores are ${\text{f}}_{{{\text{ik}}}} = \smallint \phi_{{\text{k}}} ({\text{t}})[\nu_{{\text{i}}} ({\upalpha }) - \overline{\nu }(\alpha )]{\text{d}}\alpha ,\; {\text{k}} = 1,2, \ldots ,{\text{K}}$. The number of PCs ${\text{K}}$ can be selected such that the majority, e.g. 90%, of the variance is explained by (2).

The functional PCs are orthonormal, i.e., $\smallint \phi_{{\text{j}}} (\alpha )\phi_{{\text{k}}} (\alpha ){\text{d}}\alpha = 0$ if ${\text{j}} \ne {\text{k}}$ and $1$ of ${\text{j}} = {\text{k}}$, and they explains the most proportion of variance in the data in descending order. In other words, the first PC score ${\text{f}}_{{{\text{i}}1}} ,{\text{ i}} = 1,2, \ldots {\text{n}}$ maximizes the sample variance among the inner products between the data and all functionals $\xi$’s subject to the constraint $\smallint \xi (\alpha )^{2} {\text{d}}\alpha = 1$; the second PC score ${\text{f}}_{{{\text{i}}2}} ,{\text{ i}} = 1,2, \ldots {\text{n}}$ maximizes the remaining variance among the inner products of the data and all functionals $\xi$’s subject to the constraint $\smallint \xi (\alpha )^{2} {\text{d}}\alpha = 1$ and $\smallint \xi (\alpha )\phi_{1} (\alpha ){\text{d}}\alpha = 0$, and so on. The proportion of variance that is explained by ${\text{f}}_{{{\text{ik}}}} ,\; 1 \le {\text{i}} \le {\text{n}}$ is $\lambda_{{\text{k}}} /\sum\nolimits_{{{\text{k}} = 1}}^{\infty } {\lambda_{{\text{k}}} }$. In the estimation, we regularize the functional PCs and impose roughness penalty to the sample covariance function, measured by the norm of second-order derivative of the function. Details of functional PCA can be found in Ref.^25^.

### Functional generalized linear model (functional GLM).

Assume the binary response ${\text{y}}_{{\text{i}}} \sim {\text{Bernoulli}}\;(p_{{\text{i}}} )$, where ${\text{p}}_{{\text{i}}}$ is the probability that the molecule ${\text{i}}$ contains the functional group. Denote the logit function of ${\text{p}}_{{\text{i}}}$ as ${\text{l}}_{{\text{i}}} = \log \left( {\frac{{{\text{p}}_{{\text{i}}} }}{{1 - {\text{p}}_{{\text{i}}} }}} \right)$ and assume that ${\text{l}}_{{\text{i}}}$. is a linear function of $\nu_{{\text{i}}} (\alpha )$. That is

$$ {\text{l}}_{{\text{i}}} = \beta_{0} + \smallint \beta (\alpha )\left[ {\hat{\nu }_{i} (\alpha ) - \overline{\nu }(\alpha )} \right]{\text{d}}\alpha , $$

where the intercept $\beta_{0}$ and the functional parameter $\beta (\alpha )$ are unknown quantity and coefficient functionals of interest. Represent $\beta (\alpha ) = \mathop \sum \limits_{{{\text{k}} = 1}}^{\infty } \beta_{{\text{k}}} \phi_{{\text{k}}} (\alpha )$. According to (1), the above linear model can be written as

3 $$ {\text{l}}_{{\text{i}}} = {\upbeta }_{0} + \mathop \sum \limits_{{{\text{k}} = 1}}^{{\text{K}}} {\upbeta }_{{\text{k}}} {\text{f}}_{{{\text{ik}}}} , $$

where the unknown coefficients $\beta_{0} ,\beta_{1} , \ldots ,\beta_{{\text{K}}}$ can be estimated by the maximum likelihood estimation (MLE) for the generalized linear model^26^. The above logistic regression can be extended to multiclass cases. Details are omitted and interested readers are referred to Ref.^27^.

For a new molecule with IR spectrum $\nu^{{ \star }} (\alpha )$, the probability ${\text{p}}^{{ \star }}$ can be estimated by ${\hat{\text{p}}}^{{ \star }} = \frac{{\exp ({\hat{\text{l}}}^{{ \star }} )}}{{1 + \exp ({\hat{\text{l}}}^{{ \star }} )}}$, where ${\hat{\text{l}}}^{{ \star }} = \hat{\beta }_{0} + \sum\nolimits_{{{\text{k}} = 1}}^{{\text{K}}} {\hat{\beta }_{{\text{k}}} {\text{f}}_{{\text{k}}}^{{ \star }} }$, where $\hat{\beta }_{{\text{k}}}$ is the MLE of $\beta_{{\text{k}}}$, and ${\text{f}}_{{\text{k}}}^{{ \star }}$ are the PC scores of the new molecule, ${\text{k}} = 1,\;2, \ldots ,{\text{K}}$. If ${\hat{\text{p}}}^{{ \star }}$ exceeds a given threshold such as 0.5, the molecule is classified as having the functional group.

Comparing to the high-dimensional multivariate classification with ${\text{p}} = 876$ predictors, the functional GLM represents the absorbance by a small number of orthogonal basis functions enabling statistical and computational efficiency. More importantly, the GLM associates the functional groups with features in the IR spectra in terms of the functional components along the whole range, while the multivariate classification treats absorbance at each wavenumber as a predictor and trains the model by assuming conditions such as sparsity.

### Classification performance metrics

In evaluating the performance of classification results we obtain the confusion matrix as shown in Table 2 by comparing the predicted labels against the ground truth from the test set.

**Table 2:** Confusion matrix of a classification problem.

	Ground truth		
		True	False
Prediction	True	True positive (TP)	False positive (FP)
	False	False negative (FN)	True negative (TN)

Let

$$ FPR = \frac{FP}{{FP + TN}}, \quad FNR = \frac{FN}{{FN + TP}}. $$

The Receiver Operating Characteristic Curve (ROC) is a curve plotting *1-FNR* (a.k.a., sensitivity) against the *FPR* (a.k.a., 1-specificity). For each classification model we report the Area Under the Curve (AUC) and use AUC as an evaluation metric of the algorithm performance.

## Results and discussion

In this study, we train functional GLM models and evaluate its capability to fingerprint the functional groups including amide, aniline, benzene and piperidine in the IR spectrum of a molecule. Parameters for the GLM are estimated from the IR spectral data and functional groups information of the training set to train the model. The constructed model is then used to predict the functional groups from only IR spectra data of a testing dataset. We evaluate the accuracy of the models by comparing the prediction with the ground truth in the testing set. The functional data analysis is implemented using the R package fda.usc^28^ and the ROC curve and AUC are obtained from R package pROC^29^.

We investigate two scenarios. Scenario one considers potential interactions which may arise from the simultaneous existence of multiple functional groups reflected by distinct IR spectral patterns. We classify the testing molecules into 8 nonoverlapping groups. Whereas in scenario two, the model is trained to predict if a molecule contains the constituent functional groups, where compounds with simultaneous presence of multiple functional groups is also counted.

### Feature representation: functional PCA

**Figure 4:**
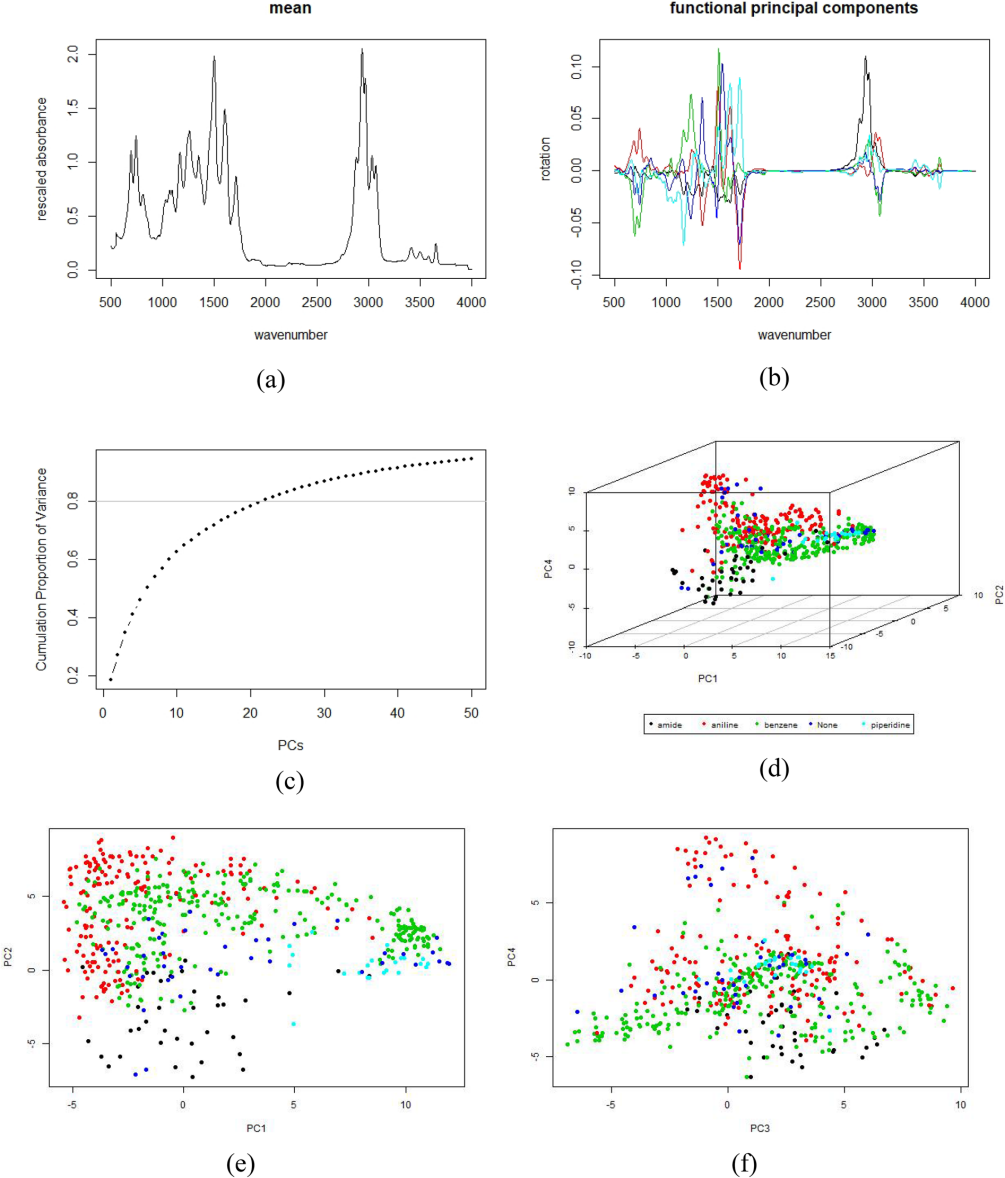
Functional PCA results: (**a**) the mean function of the rescaled absorbance; (**b**) the first five functional principal components; (**c**) the cumulative proportion of variance explained by the first 50 functional PCs; (**d**) the 3D plot of PC1, PC2 and PC4 scores of the compounds consisting of at most one functional groups of interest; (**e**) PC1 and PC2 scores of the compounds consisting of at most one functional groups of interest; (**f**) PC1 and PC2 scores of the compounds consisting of at most one functional groups of interest .

We first construct the regularized functional PCs basis from the training set. The mean function and the leading PCs are presented in Fig. 4.

From Fig. 4, one can see the following. First, as shown in Fig, 3, the IR spectral data in each group is very diverse and has various patterns. Accordingly, in panel (c) of Fig. 4, the proportion of variance explained by the functional PCs increases slowly as more PCs are considered, where the first functional PC explains nearly 20% of the overall variance, with the second describing 10%. Over 80% of the total variance may be considered with the first 22 PCs. In addition, in panel (d) to (f) we present the leading 4 PC scores of compounds with no more than one functional groups of interest. The first four PC scores alone does not partition the four groups; however, we can observe a general trend in which, compounds with amide tend to have small scores of PC1, PC2 and PC4, and high scores in PC 3; anilines tend to have high scores in PC2, while piperidine containing compounds are clustered within a narrow range of PC2 and PC4 with high PC1 scores.

### Prediction for appearance of functional groups from IR spectra

Four functional GLMs are constructed to predict the following responses from the IR spectra data and are assigned as a specific model respectively.

Model 1: whether the molecule has an *amide* functional group.
Model 2: whether the molecule has an *aniline* functional group.
Model 3: whether the molecule has a *benzene* functional group.
Model 4: whether the molecule has a *piperidine* functional group.

**Figure 5:**
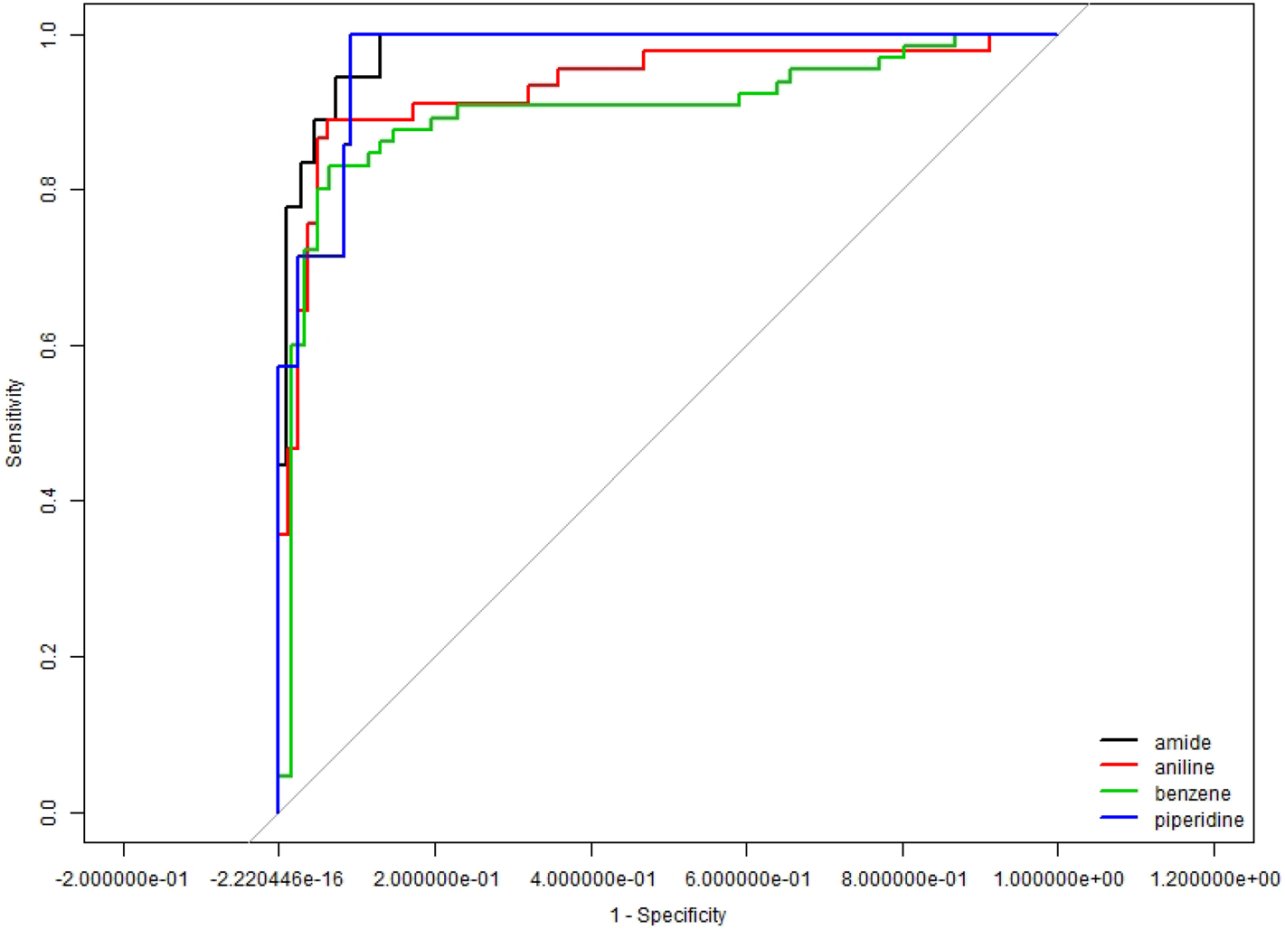
ROC curves for models 1.1 to 1.4.

In constructing the functional GLM models, we take ${\text{K}} = 22$, which is the minimum number of PCs such that at least 80% of variance is explained. In Model 4, because the response is imbalanced with only 10 piperidine out of 478 training molecules, we perform an oversampling adjustment before estimating the model. That is, the 10 observations in the training set are resampled and reused such that there are 100 molecules in the training set are piperidine. The ROC curves for Model 1 to Model 4 are presented in the area under the ROC curve on Fig. 5 are shown in the Table 5.

We present the confusion matrices from the testing set of Models 1–4 (AUCs presented in Table 3) in Tables 4, 5, 6, 7 respectively, with a threshold probability of 0.5. The rates of correctly identifying the corresponding functional groups are 96.03%, 90.48%, 87.3%, and 96.03% respectively, with an average accuracy rate of 92.5%.

**Table 3:** AUC of models 1–4.

	1—Amide	2—Aniline	3—Benzene	4—Piperidine
AUC	0.9815	0.9336	0.9019	0.9712

**Table 4:** Confusion matrix for Model 1—predicting appearance of amide.

1—Amide		Prediction	
		False	True
Ground truth	False	107	1
	True	4	14

**Table 5:** Confusion matrix for Model 2—predicting appearance of aniline.

2—Aniline		Prediction	
		False	True
Ground truth	False	74	7
	True	5	40

**Table 6:** Confusion matrix for Model 3—predicting appearance of benzene.

3—Benzene		Prediction	
		False	True
Ground truth	False	58	6
	True	13	50

**Table 7:** Confusion matrix for Model 4—predicting appearance of piperidine.

4—Piperidine		Prediction	
		False	True
Ground truth	False	115	4
	True	0	8

### Joint classification into non-overlapping groups.

We combine the four binary classification results described in Tables 4, 5, 6, 7 and group the data as shown in Table 1. The confusion matrix obtained from prediction in the 126 testing molecules is summarized in Table 8.

**Table 8:** Confusion matrix of the classification in the testing set.

		Prediction								
		Amide only	Aniline only	Benzene only	Piperidine only	Amide and aniline	Amide and benzene	Aniline and benzene	Benzene and piperidine	None
Ground truth	Amide only	5	0	0	1	1	0	0	0	0
	Aniline only	1	32	1	0	0	0	1	0	0
	Benzene only	0	3	49	0	0	0	1	0	1
	Piperidine only	0	0	0	4	0	0	0	0	1
	Amide and aniline	1	0	0	0	3	1	0	0	1
	Amide and benzene	1	1	0	0	1	1	0	0	1
	Aniline and benzene	0	3	0	0	0	0	1	0	0
	Benzene and piperidine	0	0	1	1	0	0	0	0	0
	None	0	0	1	2	0	0	0	0	5

From Table 8 one can find that, out of the 126 predicted molecules, 100 are classified exactly correctly, with an accuracy rate of 79.37%. Among the misclassified 26 molecules, 12 are classified with partial mistakes by incorrectly including or excluding a functional group while correctly identify another, 4 are misclassified by missing the correct functional group, 3 is misclassified by identifying a wrong functional group and 7 are misclassified by both missing the truth and identify a false. The misclassified molecules are listed in Table 9.

**Table 9:** Misclassified compounds and the classification errors.

Compound	Ground truth	Prediction
1-Adamantanecarboxamide, *N*,*N*-dimethyl-,	Amide only	Piperidine only
Acetamide, *N*-butyl-	Amide only	Amide and aniline
Azobenzene	Aniline only	Benzene
4-Amino-2,6-dichlorophenol	Aniline only	Aniline and Benzene
1,3-Diphenylguanidine	Aniline only	Amide only
Benzene, pentafluoromethyl-	Benzene only	Aniline only
Benzene, 1-fluoro-3-methyl-	Benzene only	Aniline and Benzene
Diethyl Phthalate	Benzene only	None
Phenol, 2,6-dimethoxy-	Benzene only	Aniline only
Benzene, 1,3-difluoro-	Benzene only	Aniline only
Cyclohexanamine	None	Piperidine only
Nitrous oxide	None	Benzene only
Triethanolamine	None	Piperidine only
4-Piperidinol, 2,2,6,6-tetramethyl-, 1-oxide	Piperidine only	None
4-Benzylpiperidine	Benzene and piperidine	Benzene only
4-(4-Chlorophenyl)-4-hydroxypiperidine	Benzene and piperidine	Piperidine only
Metazachlor	Amide and aniline	Amide and benzene
Acetamide, N-(2,6-dimethylphenyl)-	Amide and aniline	Amide only
4′-Bromoacetanilide	Amide and aniline	None
Benzamide	Amide and benzene	Amide only
Benzhydroxamic acid	Amide and Benzene	None
Dibucaine	amide and Benzene	Aniline only
Acetanilide, 4′-fluoro-	Amide and benzene	Amide and aniline
p-Butoxybenzylidene p-butylaniline	Aniline and benzene	Aniline only
p-Hexyloxybenzylidene p-butylaniline	Aniline and benzene	Aniline only
2(o-Aminophenyl)-benzimidazole	Aniline and benzene	Aniline only

Table 9 shows a relatively high error rate in distinguishing simultaneously appeared functional groups from the cases where only one of the functional groups of interest is present. The algorithm also has a relatively high error rate in distinguishing the aniline group and benzene, due to fact that these functional groups share common structure.

## Summary

Today, synthetic opioid analogues such as, fentanyl, have been the cause of many accidental deaths across the world, and thus the detection of low concentrations of these harmful substances at a distance via spectroscopic techniques is crucial for law enforcement. Unfortunately, new fentanyl related compounds are being created and distributed frequently with slight modifications to the functional groups present in them. Therefore, machine learning based intelligent detection schemes must be employed for intelligent detection of such molecules. Here, we applied a functional generalized linear model with smoothed functional principal component basis to classify functional groups of molecules from their IR absorption data. The result serves as a basis of the identification of fentanyl and its derivatives, which could be accomplished in future. This effort demonstrated the efficacy of a functional data analysis to identify molecules containing one or more specific functional groups from their infrared absorption spectra. The accuracy rate of classification into the 9 distinct classes is 79.4%. The average rate of accurately identifying the four functional groups is 92.5%. Continued efforts on this model will seek to also expand the utility from gas phase compound analysis to solid phase, with spectral properties of fentanyl and its analogues included. Such expansions will require additional consideration.

## Supplementary information

Supplementary Appendix A.

Supplementary Appendix B.
