# Supplementary material for: High accuracy machine learning identification of fentanyl-relevant molecular compound classification via constituent functional group analysis
Source: Sci Rep. 2020 Aug 11;10:13569. doi: 10.1038/s41598-020-70471-7 (PMC7419312; doi:10.1038/s41598-020-70471-7)
Supplement: Supplementary file 1 — Supplementary Appendix A. [file 41598_2020_70471_MOESM1_ESM.docx]

**Supplementary Information- Appendix A**

**Title: High Accuracy Machine Learning Identification of Fentanyl-Relevant Molecular Compound Classification *via* Constituent Functional Group Analysis**

**Authors:**  Mengyu Xu,^1,4^ Chun-Hung Wang,^2^ Anthony C. Terracciano,^3,4^ Artem E. Masunov,^2,5-8^ and Subith S. Vasu^3,4, *^

**Affiliations:**

^1^Statistics and Data Science, University of Central Florida, 4000 Central Florida Blvd, Orlando Fl, 32816, USA

^2^NanoScience Technology Center, University of Central Florida, 12424 Research Parkway, Orlando Fl, 32826, USA

^3^Mechanical and Aerospace Engineering, University of Central Florida, 4000 Central Florida Blvd, Orlando Fl, 32816, USA

^4^Center for Advanced Turbomachinery and Energy Research, University of Central Florida, 4000 Central Florida Blvd, Orlando Fl, 32816, USA

^5^School of Modeling, Simulation, and Training, University of Central Florida, 3100 Technology Parkway, Orlando, FL 32816, USA

^6^Department of Chemistry, University of Central Florida, 4111 Libra Dr., Orlando, FL 32816, USA

^7^South Ural State University, Lenin pr. 76, Chelyabinsk 454080, Russia

^8^National Research Nuclear University MEPhI, Kashirskoye shosse 31, Moscow, 115409, Russia

*Corresponding: subith@ucf.edu

In this appendix we include the source and group of the molecules included in the dataset.

| names | links | group |
| --- | --- | --- |
| Lidocaine | https://webbook.nist.gov/cgi/cbook.cgi?ID=C137586 | simult_Amide&benzene |
| Formamide, N,N-dimethyl- | https://webbook.nist.gov/cgi/cbook.cgi?ID=C68122 | amide |
| Acetamide | https://webbook.nist.gov/cgi/cbook.cgi?ID=C60355 | amide |
| Caprolactam | https://webbook.nist.gov/cgi/cbook.cgi?ID=C105602 | amide |
| Acetamide, N,N-dimethyl- | https://webbook.nist.gov/cgi/cbook.cgi?ID=C127195 | amide |
| Dimethoate | https://webbook.nist.gov/cgi/cbook.cgi?ID=C60515 | amide |
| Formamide | https://webbook.nist.gov/cgi/cbook.cgi?ID=C75127 | amide |
| N-Methylacetamide | https://webbook.nist.gov/cgi/cbook.cgi?ID=C79163 | amide |
| Benzamide | https://webbook.nist.gov/cgi/cbook.cgi?ID=C55210 | simult_Amide&benzene |
| Benzamide, N,N-dimethyl- | https://webbook.nist.gov/cgi/cbook.cgi?ID=C611745 | simult_Amide&benzene |
| Carbazepine | https://webbook.nist.gov/cgi/cbook.cgi?ID=C298464 | simult_Amide&benzene |
| Formamide, N-methyl- | https://webbook.nist.gov/cgi/cbook.cgi?ID=C123397 | amide |
| Promethazine | https://webbook.nist.gov/cgi/cbook.cgi?ID=C60877 | benzene |
| Phosphamidon | https://webbook.nist.gov/cgi/cbook.cgi?ID=C13171216 | amide |
| Diphenamid | https://webbook.nist.gov/cgi/cbook.cgi?ID=C957517 | simult_Amide&benzene |
| Acetamide, N-ethyl- | https://webbook.nist.gov/cgi/cbook.cgi?ID=C625503 | amide |
| Formamide, N,N-diethyl- | https://webbook.nist.gov/cgi/cbook.cgi?ID=C617845 | amide |
| Captan | https://webbook.nist.gov/cgi/cbook.cgi?ID=C133062 | amide |
| Nicotinamide | https://webbook.nist.gov/cgi/cbook.cgi?ID=C98920 | amide |
| Acetamide, N,N-diethyl- | https://webbook.nist.gov/cgi/cbook.cgi?ID=C685916 | amide |
| Dimethoate oxygen analog | https://webbook.nist.gov/cgi/cbook.cgi?ID=C1113026 | amide |
| Acephate | https://webbook.nist.gov/cgi/cbook.cgi?ID=C30560191 | amide |
| Acetamide, N-butyl- | https://webbook.nist.gov/cgi/cbook.cgi?ID=C1119499 | amide |
| Propachlor | https://webbook.nist.gov/cgi/cbook.cgi?ID=C1918167 | simult_Amide&aniline |
| Pentanamide | https://webbook.nist.gov/cgi/cbook.cgi?ID=C626971 | amide |
| Acetamide, N,N-dibutyl- | https://webbook.nist.gov/cgi/cbook.cgi?ID=C1563902 | amide |
| Cyanamide, dimethyl- | https://webbook.nist.gov/cgi/cbook.cgi?ID=C1467794 | None |
| Pyrazinamide | https://webbook.nist.gov/cgi/cbook.cgi?ID=C98964 | amide |
| Pentadecylamine | https://webbook.nist.gov/cgi/cbook.cgi?ID=C2570265 | None |
| Carboxin | https://webbook.nist.gov/cgi/cbook.cgi?ID=C5234684 | simult_Amide&aniline |
| Butachlor | https://webbook.nist.gov/cgi/cbook.cgi?ID=C23184669 | simult_Amide&aniline |
| Ethanamine, N-ethyl-N-nitroso- | https://webbook.nist.gov/cgi/cbook.cgi?ID=C55185 | None |
| Deet | https://webbook.nist.gov/cgi/cbook.cgi?ID=C134623 | simult_Amide&benzene |
| N-tert-Butylacetamide | https://webbook.nist.gov/cgi/cbook.cgi?ID=C762845 | amide |
| 1-Adamantanecarboxamide, N,N-dimethyl-, | https://webbook.nist.gov/cgi/cbook.cgi?ID=C1502007 | amide |
| Ethamivan | https://webbook.nist.gov/cgi/cbook.cgi?ID=C304847 | simult_Amide&benzene |
| Thiram | https://webbook.nist.gov/cgi/cbook.cgi?ID=C137268 | None |
| Hexamethylphosphoric triamide | https://webbook.nist.gov/cgi/cbook.cgi?ID=C680319 | None |
| Famphur | https://webbook.nist.gov/cgi/cbook.cgi?ID=C52857 | benzene |
| Benzenesulfonamide, N-butyl- | https://webbook.nist.gov/cgi/cbook.cgi?ID=C3622842 | benzene |
| Benzenesulfonamide, 4-methyl- | https://webbook.nist.gov/cgi/cbook.cgi?ID=C70553 | benzene |
| Acetamide, N-(phenylmethyl)- | https://webbook.nist.gov/cgi/cbook.cgi?ID=C588465 | simult_Amide&benzene |
| Crufomate | https://webbook.nist.gov/cgi/cbook.cgi?ID=C299865 | benzene |
| N-Ethylformamide | https://webbook.nist.gov/cgi/cbook.cgi?ID=C627452 | amide |
| Oryzalin | https://webbook.nist.gov/cgi/cbook.cgi?ID=C19044883 | simult_aniline&benzene |
| m-Aminobenzenesulfonamide | https://webbook.nist.gov/cgi/cbook.cgi?ID=C98180 | simult_aniline&benzene |
| Monocrotophos | https://webbook.nist.gov/cgi/cbook.cgi?ID=C6923224 | amide |
| Oxamyl | https://webbook.nist.gov/cgi/cbook.cgi?ID=C23135220 | amide |
| Benzoyl hydrazine | https://webbook.nist.gov/cgi/cbook.cgi?ID=C613945 | simult_Amide&benzene |
| Metazachlor | https://webbook.nist.gov/cgi/cbook.cgi?ID=C67129082 | simult_Amide&aniline |
| Acetanilide, 4'-fluoro- | https://webbook.nist.gov/cgi/cbook.cgi?ID=C351837 | simult_Amide&benzene |
| 4-Chlorobenzamide | https://webbook.nist.gov/cgi/cbook.cgi?ID=C619567 | simult_Amide&benzene |
| Formamide, N-(2-phenylethyl)- | https://webbook.nist.gov/cgi/cbook.cgi?ID=C23069990 | simult_Amide&benzene |
| Acetamide, N-(4-methoxyphenyl)-N-methyl- | https://webbook.nist.gov/cgi/cbook.cgi?ID=C35813388 | simult_Amide&aniline |
| 2',4'-Acetoxylidide | https://webbook.nist.gov/cgi/cbook.cgi?ID=C2050433 | simult_Amide&aniline |
| p-Fluorobenzamide | https://webbook.nist.gov/cgi/cbook.cgi?ID=C824759 | simult_Amide&benzene |
| Morpholine, 4-(2-benzothiazolylthio)- | https://webbook.nist.gov/cgi/cbook.cgi?ID=C102772 | benzene |
| m-Aminobenzamide | https://webbook.nist.gov/cgi/cbook.cgi?ID=C3544249 | simult_Amide&aniline |
| Acetamide, N,N-bis(1-methylethyl)- | https://webbook.nist.gov/cgi/cbook.cgi?ID=C759228 | amide |
| N-Benzylformamide | https://webbook.nist.gov/cgi/cbook.cgi?ID=C6343540 | simult_Amide&benzene |
| 4-Acetamidoantipyrene | https://webbook.nist.gov/cgi/cbook.cgi?ID=C83158 | simult_Amide&aniline |
| N,N-Dimethyltrifluoroacetamide | https://webbook.nist.gov/cgi/cbook.cgi?ID=C1547871 | amide |
| Benzenesulfonamide, N,N,4-trimethyl- | https://webbook.nist.gov/cgi/cbook.cgi?ID=C599699 | benzene |
| Benzenesulfonamide, N,4-dimethyl- | https://webbook.nist.gov/cgi/cbook.cgi?ID=C640619 | benzene |
| Benzeneacetamide, .alpha.-ethyl- | https://webbook.nist.gov/cgi/cbook.cgi?ID=C90266 | simult_Amide&benzene |
| Butylformamide | https://webbook.nist.gov/cgi/cbook.cgi?ID=C871716 | amide |
| (.alpha.-Bromoisovaleryl)urea | https://webbook.nist.gov/cgi/cbook.cgi?ID=C496673 | amide |
| Methanesulfonamide, N,N-dimethyl- | https://webbook.nist.gov/cgi/cbook.cgi?ID=C918058 | None |
| Dodecanamide, N,N-diethyl- | https://webbook.nist.gov/cgi/cbook.cgi?ID=C3352872 | amide |
| Acetamide, 2-chloro-N,N-diethyl- | https://webbook.nist.gov/cgi/cbook.cgi?ID=C2315368 | amide |
| Benzenesulfonamide, 2-methyl- | https://webbook.nist.gov/cgi/cbook.cgi?ID=C88197 | benzene |
| o-Fluorobenzamide | https://webbook.nist.gov/cgi/cbook.cgi?ID=C445283 | simult_Amide&benzene |
| Benzenesulfonamide, N-butyl-4-methyl- | https://webbook.nist.gov/cgi/cbook.cgi?ID=C1907659 | benzene |
| N,N'-Bis(2-hydroxyethyl)dithioxamide | https://webbook.nist.gov/cgi/cbook.cgi?ID=C120865 | None |
| Furamide | https://webbook.nist.gov/cgi/cbook.cgi?ID=C609381 | amide |
| Benzhydroxamic acid | https://webbook.nist.gov/cgi/cbook.cgi?ID=C495181 | simult_Amide&benzene |
| N',n-Butyl-3-amino-4-methoxybenzenesulfon | https://webbook.nist.gov/cgi/cbook.cgi?ID=C80228 | aniline |
| 2-Propenamide, N-(1,1-dimethyl-3-oxobutyl)- | https://webbook.nist.gov/cgi/cbook.cgi?ID=C2873974 | amide |
| N-t-Butylacrylamide | https://webbook.nist.gov/cgi/cbook.cgi?ID=C107584 | amide |
| Bicyclo[2.2.1]-5-heptene-2-carboxamide | https://webbook.nist.gov/cgi/cbook.cgi?ID=C95170 | amide |
| N,N-Dimethylforamide | https://webbook.nist.gov/cgi/cbook.cgi?ID=C4472417 | None |
| Acetamide, N-[2-(trifluoromethyl)phenyl]- | https://webbook.nist.gov/cgi/cbook.cgi?ID=C344627 | amide |
| N-(.alpha.-Methylbenzyl)-formamide | https://webbook.nist.gov/cgi/cbook.cgi?ID=C6948012 | amide |
| Acetanilide, 2-chloro-4'-nitro- | https://webbook.nist.gov/cgi/cbook.cgi?ID=C17329872 | simult_Amide&aniline |
| 4-Chlorobenzenesulfonamide | https://webbook.nist.gov/cgi/cbook.cgi?ID=C98646 | benzene |
| 5-Amino-4-methoxybenzamide | https://webbook.nist.gov/cgi/cbook.cgi?ID=C17481275 | simult_Amide&aniline |
| p-Trifluoromethylacetanilide | https://webbook.nist.gov/cgi/cbook.cgi?ID=C349973 | simult_Amide&aniline |
| Benzoic acid, 2-amino-, methyl ester | https://webbook.nist.gov/cgi/cbook.cgi?ID=C134203 | aniline |
| Benzocaine | https://webbook.nist.gov/cgi/cbook.cgi?ID=C94097 | aniline |
| [1,1'-Biphenyl]-2-amine | https://webbook.nist.gov/cgi/cbook.cgi?ID=C90415 | simult_aniline&benzene |
| Benzenamine, 2,4,6-tribromo- | https://webbook.nist.gov/cgi/cbook.cgi?ID=C147820 | aniline |
| 4,4'-Methylenedianiline | https://webbook.nist.gov/cgi/cbook.cgi?ID=C101779 | aniline |
| Triphenylamine | https://webbook.nist.gov/cgi/cbook.cgi?ID=C603349 | aniline |
| Benzenamine, 2,4-dichloro- | https://webbook.nist.gov/cgi/cbook.cgi?ID=C554007 | aniline |
| p-N,N-Dimethylaminobenzaldehyde | https://webbook.nist.gov/cgi/cbook.cgi?ID=C100107 | aniline |
| Benzoic acid, 2-amino-, ethyl ester | https://webbook.nist.gov/cgi/cbook.cgi?ID=C87252 | aniline |
| [1,1'-Biphenyl]-4-amine | https://webbook.nist.gov/cgi/cbook.cgi?ID=C92671 | simult_aniline&benzene |
| Benfluralin | https://webbook.nist.gov/cgi/cbook.cgi?ID=C1861401 | aniline |
| Prowl | https://webbook.nist.gov/cgi/cbook.cgi?ID=C40487421 | aniline |
| Benzenamine, N,N,3-trimethyl- | https://webbook.nist.gov/cgi/cbook.cgi?ID=C121722 | aniline |
| Benzenamine, 2,3-dichloro- | https://webbook.nist.gov/cgi/cbook.cgi?ID=C608275 | aniline |
| Benzenamine, 2-methoxy- | https://webbook.nist.gov/cgi/cbook.cgi?ID=C90040 | aniline |
| Benzidine | https://webbook.nist.gov/cgi/cbook.cgi?ID=C92875 | aniline |
| Benzenamine, 2-ethyl- | https://webbook.nist.gov/cgi/cbook.cgi?ID=C578541 | aniline |
| Benzenamine, 2,5-dimethyl- | https://webbook.nist.gov/cgi/cbook.cgi?ID=C95783 | aniline |
| O-Bromoaniline | https://webbook.nist.gov/cgi/cbook.cgi?ID=C615361 | aniline |
| Ethanone, 1-(3-aminophenyl)- | https://webbook.nist.gov/cgi/cbook.cgi?ID=C99036 | aniline |
| m-Anisidine | https://webbook.nist.gov/cgi/cbook.cgi?ID=C536903 | aniline |
| Benzenamine, 2,4,6-trimethyl- | https://webbook.nist.gov/cgi/cbook.cgi?ID=C88051 | aniline |
| Benzenamine, 3,4-dimethyl- | https://webbook.nist.gov/cgi/cbook.cgi?ID=C95647 | aniline |
| Benzenamine, N,N-diethyl-4-methyl- | https://webbook.nist.gov/cgi/cbook.cgi?ID=C613489 | aniline |
| Benzenamine, N,N-dimethyl-3-nitro- | https://webbook.nist.gov/cgi/cbook.cgi?ID=C619318 | aniline |
| Benzenamine, 3,5-dichloro- | https://webbook.nist.gov/cgi/cbook.cgi?ID=C626437 | aniline |
| Benzenamine, 3-fluoro- | https://webbook.nist.gov/cgi/cbook.cgi?ID=C372190 | aniline |
| Benzenamine, 5-chloro-2-methyl- | https://webbook.nist.gov/cgi/cbook.cgi?ID=C95794 | aniline |
| p-Bromoaniline | https://webbook.nist.gov/cgi/cbook.cgi?ID=C106401 | aniline |
| Benzenamine, 3,5-dimethyl- | https://webbook.nist.gov/cgi/cbook.cgi?ID=C108690 | aniline |
| Benzenamine, 4-ethoxy- | https://webbook.nist.gov/cgi/cbook.cgi?ID=C156434 | aniline |
| Benzenamine, 2,3-dimethyl- | https://webbook.nist.gov/cgi/cbook.cgi?ID=C87592 | aniline |
| Benzenamine, 3-chloro-4-methyl- | https://webbook.nist.gov/cgi/cbook.cgi?ID=C95749 | aniline |
| Benzenamine, 3-(trifluoromethyl)- | https://webbook.nist.gov/cgi/cbook.cgi?ID=C98168 | aniline |
| Benzenamine, 2,6-diethyl- | https://webbook.nist.gov/cgi/cbook.cgi?ID=C579668 | aniline |
| Benzenamine, 2-chloro-6-methyl- | https://webbook.nist.gov/cgi/cbook.cgi?ID=C87638 | aniline |
| Benzenamine, 3-chloro-2-methyl- | https://webbook.nist.gov/cgi/cbook.cgi?ID=C87605 | aniline |
| 4'-Methoxy-2'-hydroxybenzylidene-4-n-butylaniline | https://webbook.nist.gov/cgi/cbook.cgi?ID=C30633944 | simult_aniline&benzene |
| Benzenamine, 2-fluoro- | https://webbook.nist.gov/cgi/cbook.cgi?ID=C348549 | aniline |
| Benzenamine, 2-chloro-4-methyl- | https://webbook.nist.gov/cgi/cbook.cgi?ID=C615656 | aniline |
| Benzenamine, N-ethyl-3-methyl- | https://webbook.nist.gov/cgi/cbook.cgi?ID=C102272 | aniline |
| 4-t-Butylbenzeneamine | https://webbook.nist.gov/cgi/cbook.cgi?ID=C769926 | aniline |
| 2-Aminobenzophenone | https://webbook.nist.gov/cgi/cbook.cgi?ID=C2835770 | simult_aniline&benzene |
| 3-Ethylaniline | https://webbook.nist.gov/cgi/cbook.cgi?ID=C587020 | aniline |
| Benzenamine, N-butyl- | https://webbook.nist.gov/cgi/cbook.cgi?ID=C1126789 | aniline |
| 4-Phenylazoaniline | https://webbook.nist.gov/cgi/cbook.cgi?ID=C60093 | aniline |
| Benzenamine, 2-ethoxy- | https://webbook.nist.gov/cgi/cbook.cgi?ID=C94702 | aniline |
| 3-Aminobenzophenone | https://webbook.nist.gov/cgi/cbook.cgi?ID=C2835781 | simult_aniline&benzene |
| 3,3'-Dichlorobenzidine | https://webbook.nist.gov/cgi/cbook.cgi?ID=C91941 | aniline |
| Benzenamine, 2,6-dibromo- | https://webbook.nist.gov/cgi/cbook.cgi?ID=C608300 | aniline |
| Benzenamine, 4-butyl- | https://webbook.nist.gov/cgi/cbook.cgi?ID=C104132 | aniline |
| Benzenamine, 2,4-dibromo- | https://webbook.nist.gov/cgi/cbook.cgi?ID=C615576 | aniline |
| Benzenamine, 3-iodo- | https://webbook.nist.gov/cgi/cbook.cgi?ID=C626017 | aniline |
| N-Ethyl-p-toluidine | https://webbook.nist.gov/cgi/cbook.cgi?ID=C622571 | aniline |
| Benzenamine, N,3-dimethyl- | https://webbook.nist.gov/cgi/cbook.cgi?ID=C696446 | aniline |
| Benzenamine, N,N-diethyl-3-methyl- | https://webbook.nist.gov/cgi/cbook.cgi?ID=C91678 | aniline |
| Benzenamine, 2-methyl-5-nitro- | https://webbook.nist.gov/cgi/cbook.cgi?ID=C99558 | aniline |
| Benzenamine, 2,6-bis(1-methylethyl)- | https://webbook.nist.gov/cgi/cbook.cgi?ID=C24544045 | aniline |
| Benzenamine, N-methyl-2,4-dinitro- | https://webbook.nist.gov/cgi/cbook.cgi?ID=C2044884 | aniline |
| 2-Bromo-4-chloroaniline | https://webbook.nist.gov/cgi/cbook.cgi?ID=C873381 | aniline |
| Benzenethiol, 2-amino- | https://webbook.nist.gov/cgi/cbook.cgi?ID=C137075 | aniline |
| Benzenamine, 3,5-bis(trifluoromethyl)- | https://webbook.nist.gov/cgi/cbook.cgi?ID=C328745 | aniline |
| N-Methyl-O-toluidine | https://webbook.nist.gov/cgi/cbook.cgi?ID=C611212 | aniline |
| Benzenamine, N,N-diethyl-2-methyl- | https://webbook.nist.gov/cgi/cbook.cgi?ID=C606462 | aniline |
| Benzenamine, 3-(methylthio)- | https://webbook.nist.gov/cgi/cbook.cgi?ID=C1783819 | aniline |
| 2,3,5,6-Tetrafluoroaniline | https://webbook.nist.gov/cgi/cbook.cgi?ID=C700174 | aniline |
| Fluchloralin | https://webbook.nist.gov/cgi/cbook.cgi?ID=C33245395 | aniline |
| Benzenamine, 2-(trifluoromethyl)- | https://webbook.nist.gov/cgi/cbook.cgi?ID=C88175 | aniline |
| Benzenamine, 3,4,5-trichloro- | https://webbook.nist.gov/cgi/cbook.cgi?ID=C634913 | aniline |
| 4,5-Dichloro-2-nitroaniline | https://webbook.nist.gov/cgi/cbook.cgi?ID=C6641641 | aniline |
| 1,4-Benzenediamine, N-(1-methylethyl)-N'-phenyl- | https://webbook.nist.gov/cgi/cbook.cgi?ID=C101724 | aniline |
| Benzenamine, N-(1-methylethyl)- | https://webbook.nist.gov/cgi/cbook.cgi?ID=C768525 | aniline |
| Acetamide, N-butyl-N-phenyl- | https://webbook.nist.gov/cgi/cbook.cgi?ID=C91496 | simult_Amide&aniline |
| Benzenamine, 2-ethyl-6-methyl- | https://webbook.nist.gov/cgi/cbook.cgi?ID=C24549062 | aniline |
| 4-Aminobenzotrifluoride | https://webbook.nist.gov/cgi/cbook.cgi?ID=C455141 | aniline |
| Benzenamine, 2-bromo-4-methyl- | https://webbook.nist.gov/cgi/cbook.cgi?ID=C583686 | aniline |
| 4-Benzloxyaniline | https://webbook.nist.gov/cgi/cbook.cgi?ID=C6373462 | simult_aniline&benzene |
| Benzenamine, 3-phenoxy- | https://webbook.nist.gov/cgi/cbook.cgi?ID=C3586127 | simult_aniline&benzene |
| Benzenamine, 4-fluoro-2-methyl- | https://webbook.nist.gov/cgi/cbook.cgi?ID=C452711 | aniline |
| Benzenamine, 4-methyl-2-nitro- | https://webbook.nist.gov/cgi/cbook.cgi?ID=C89623 | aniline |
| Benzenamine, 4-methyl-3-nitro- | https://webbook.nist.gov/cgi/cbook.cgi?ID=C119324 | aniline |
| Propanenitrile, 3-(phenylamino)- | https://webbook.nist.gov/cgi/cbook.cgi?ID=C1075769 | aniline |
| 5-Chloro-2-hydroxyaniline | https://webbook.nist.gov/cgi/cbook.cgi?ID=C95852 | aniline |
| Benzenamine, 2,3,4-trichloro- | https://webbook.nist.gov/cgi/cbook.cgi?ID=C634673 | aniline |
| 4-Aminostyrene | https://webbook.nist.gov/cgi/cbook.cgi?ID=C1520214 | aniline |
| Benzonitrile, 2-amino- | https://webbook.nist.gov/cgi/cbook.cgi?ID=C1885296 | aniline |
| Benzenamine, N-ethyl-2-methyl- | https://webbook.nist.gov/cgi/cbook.cgi?ID=C94688 | aniline |
| Benzenamine, N,N-bis(2-chloroethyl)- | https://webbook.nist.gov/cgi/cbook.cgi?ID=C553275 | aniline |
| o-Toluidine, 5-isopropyl- | https://webbook.nist.gov/cgi/cbook.cgi?ID=C2051538 | aniline |
| Benzenamine, 2-methyl-4-nitro- | https://webbook.nist.gov/cgi/cbook.cgi?ID=C99525 | aniline |
| N-Allylaniline | https://webbook.nist.gov/cgi/cbook.cgi?ID=C589093 | aniline |
| p-Aminophenylacetic acid | https://webbook.nist.gov/cgi/cbook.cgi?ID=C1197553 | aniline |
| Aniline, 2-ethyl-N-methyl-, | https://webbook.nist.gov/cgi/cbook.cgi?ID=C1821381 | aniline |
| Benzenamine, 4-methoxy-N-methyl- | https://webbook.nist.gov/cgi/cbook.cgi?ID=C5961591 | aniline |
| 1,3-Diphenylguanidine | https://webbook.nist.gov/cgi/cbook.cgi?ID=C102067 | aniline |
| p-Anisidine, 3-nitro-, | https://webbook.nist.gov/cgi/cbook.cgi?ID=C577720 | aniline |
| Benzenamine, 4-(hexyloxy)- | https://webbook.nist.gov/cgi/cbook.cgi?ID=C39905572 | aniline |
| N-(2-Hydroxyethyl)-N-methylaniline | https://webbook.nist.gov/cgi/cbook.cgi?ID=C93903 | aniline |
| 3-Hydroxydiphenylamine | https://webbook.nist.gov/cgi/cbook.cgi?ID=C101188 | aniline |
| Benzenamine, 2,3,5,6-tetrachloro- | https://webbook.nist.gov/cgi/cbook.cgi?ID=C3481207 | aniline |
| Benzenamine, 3-methyl-4-nitro- | https://webbook.nist.gov/cgi/cbook.cgi?ID=C611052 | aniline |
| 2-Methyl-6-nitroaniline | https://webbook.nist.gov/cgi/cbook.cgi?ID=C570241 | aniline |
| 2-(4-Aminophenyl)ethylamine | https://webbook.nist.gov/cgi/cbook.cgi?ID=C13472009 | aniline |
| 1,3-Benzodioxol-5-amine | https://webbook.nist.gov/cgi/cbook.cgi?ID=C14268667 | aniline |
| 4-Bromo-3-methylaniline | https://webbook.nist.gov/cgi/cbook.cgi?ID=C6933104 | aniline |
| Aniline, N,N-diallyl- | https://webbook.nist.gov/cgi/cbook.cgi?ID=C6247003 | aniline |
| 2-Methyl-3-nitroaniline | https://webbook.nist.gov/cgi/cbook.cgi?ID=C603838 | aniline |
| 2-Methylmercaptoaniline | https://webbook.nist.gov/cgi/cbook.cgi?ID=C2987533 | aniline |
| Benzenamine, N-dodecyl- | https://webbook.nist.gov/cgi/cbook.cgi?ID=C3007747 | aniline |
| Propanenitrile, 3-(ethylphenylamino)- | https://webbook.nist.gov/cgi/cbook.cgi?ID=C148878 | aniline |
| Benzenamine, 4-chloro-2-nitro-N-phenyl- | https://webbook.nist.gov/cgi/cbook.cgi?ID=C16611157 | aniline |
| 4-Aminophenyl disulfide | https://webbook.nist.gov/cgi/cbook.cgi?ID=C722270 | aniline |
| N,N-Diethyl-N'-phenylethylenediamine | https://webbook.nist.gov/cgi/cbook.cgi?ID=C1665594 | aniline |
| 4-Amino-3-nitrobenzotrifluoride | https://webbook.nist.gov/cgi/cbook.cgi?ID=C400986 | aniline |
| Benzenamine, 2,3,4,5-tetrachloro- | https://webbook.nist.gov/cgi/cbook.cgi?ID=C634833 | aniline |
| o-(p-Toluylsulfonamido)aniline | https://webbook.nist.gov/cgi/cbook.cgi?ID=C3624906 | simult_aniline&benzene |
| Benzenamine, 2-bromo-4-nitro- | https://webbook.nist.gov/cgi/cbook.cgi?ID=C13296941 | aniline |
| 5-Amino-2-nitrobenzotrifluoride | https://webbook.nist.gov/cgi/cbook.cgi?ID=C393113 | aniline |
| 2(o-Aminophenyl)-benzimidazole | https://webbook.nist.gov/cgi/cbook.cgi?ID=C5805390 | simult_aniline&benzene |
| 4-Amino-2,6-dichlorophenol | https://webbook.nist.gov/cgi/cbook.cgi?ID=C5930289 | aniline |
| Bis(2-aminophenyl)disulfide | https://webbook.nist.gov/cgi/cbook.cgi?ID=C1141884 | aniline |
| Benzenamine, 2,4-dimethoxy- | https://webbook.nist.gov/cgi/cbook.cgi?ID=C2735048 | aniline |
| 2-Amino-5-iodotoluene | https://webbook.nist.gov/cgi/cbook.cgi?ID=C13194688 | aniline |
| 2-(o-Tolyloxy)-aniline | https://webbook.nist.gov/cgi/cbook.cgi?ID=C3840184 | simult_aniline&benzene |
| Methanesulfanilide | https://webbook.nist.gov/cgi/cbook.cgi?ID=C1197224 | aniline |
| Benzene, 1-methyl-4-(1-methylethyl)- | https://webbook.nist.gov/cgi/cbook.cgi?ID=C99876 | benzene |
| Toluene | https://webbook.nist.gov/cgi/cbook.cgi?ID=C108883 | benzene |
| Phenethyl alcohol | https://webbook.nist.gov/cgi/cbook.cgi?ID=C60128 | benzene |
| Benzene | https://webbook.nist.gov/cgi/cbook.cgi?ID=C71432 | benzene |
| Eugenol | https://webbook.nist.gov/cgi/cbook.cgi?ID=C97530 | benzene |
| Benzylalcohol | https://webbook.nist.gov/cgi/cbook.cgi?ID=C100516 | benzene |
| Ethylbenzene | https://webbook.nist.gov/cgi/cbook.cgi?ID=C100414 | benzene |
| p-Xylene | https://webbook.nist.gov/cgi/cbook.cgi?ID=C106423 | benzene |
| Benzene, 1,2-dimethyl- | https://webbook.nist.gov/cgi/cbook.cgi?ID=C95476 | benzene |
| Benzene, 1,3-dimethyl- | https://webbook.nist.gov/cgi/cbook.cgi?ID=C108383 | benzene |
| Phenol | https://webbook.nist.gov/cgi/cbook.cgi?ID=C108952 | benzene |
| Cyclohexane | https://webbook.nist.gov/cgi/cbook.cgi?ID=C110827 | None |
| Biphenyl | https://webbook.nist.gov/cgi/cbook.cgi?ID=C92524 | benzene |
| Acetophenone | https://webbook.nist.gov/cgi/cbook.cgi?ID=C98862 | benzene |
| Phenol, 2-methoxy- | https://webbook.nist.gov/cgi/cbook.cgi?ID=C90051 | benzene |
| Pyridine | https://webbook.nist.gov/cgi/cbook.cgi?ID=C110861 | None |
| Benzene, 1,3,5-trimethyl- | https://webbook.nist.gov/cgi/cbook.cgi?ID=C108678 | benzene |
| 1-Allyl-3,4-dimethoxybenzene | https://webbook.nist.gov/cgi/cbook.cgi?ID=C93152 | benzene |
| Phenol, 4-methyl- | https://webbook.nist.gov/cgi/cbook.cgi?ID=C106445 | benzene |
| Benzene, propyl- | https://webbook.nist.gov/cgi/cbook.cgi?ID=C103651 | benzene |
| Benzene, 1,2,4-trimethyl- | https://webbook.nist.gov/cgi/cbook.cgi?ID=C95636 | benzene |
| Benzene, (1-methylethyl)- | https://webbook.nist.gov/cgi/cbook.cgi?ID=C98828 | benzene |
| 1-Methyl-4-iso-propenylbenzene | https://webbook.nist.gov/cgi/cbook.cgi?ID=C1195320 | benzene |
| Benzene, butyl- | https://webbook.nist.gov/cgi/cbook.cgi?ID=C104518 | benzene |
| Benzene, 1,2,3-trimethyl- | https://webbook.nist.gov/cgi/cbook.cgi?ID=C526738 | benzene |
| Benzoic Acid | https://webbook.nist.gov/cgi/cbook.cgi?ID=C65850 | benzene |
| Cyclohexene | https://webbook.nist.gov/cgi/cbook.cgi?ID=C110838 | None |
| Benzene, chloro- | https://webbook.nist.gov/cgi/cbook.cgi?ID=C108907 | benzene |
| Benzene, 1-ethyl-3-methyl- | https://webbook.nist.gov/cgi/cbook.cgi?ID=C620144 | benzene |
| Phenol, 2-methyl- | https://webbook.nist.gov/cgi/cbook.cgi?ID=C95487 | benzene |
| Phenol, 3-methyl- | https://webbook.nist.gov/cgi/cbook.cgi?ID=C108394 | benzene |
| 1-Methyl-2-isopropylbenzene | https://webbook.nist.gov/cgi/cbook.cgi?ID=C527844 | benzene |
| 1-Allyl-4-methoxybenzene | https://webbook.nist.gov/cgi/cbook.cgi?ID=C140670 | benzene |
| Benzene, 1-ethyl-2-methyl- | https://webbook.nist.gov/cgi/cbook.cgi?ID=C611143 | benzene |
| Acetic acid, phenylmethyl ester | https://webbook.nist.gov/cgi/cbook.cgi?ID=C140114 | benzene |
| 1-Ethyl-4-methylbenzene | https://webbook.nist.gov/cgi/cbook.cgi?ID=C622968 | benzene |
| Fluoranthene | https://webbook.nist.gov/cgi/cbook.cgi?ID=C206440 | benzene |
| Benzene, 1,2,4,5-tetramethyl- | https://webbook.nist.gov/cgi/cbook.cgi?ID=C95932 | benzene |
| Benzene, methoxy- | https://webbook.nist.gov/cgi/cbook.cgi?ID=C100663 | benzene |
| Diphenylmethane | https://webbook.nist.gov/cgi/cbook.cgi?ID=C101815 | benzene |
| 1-Methyl-3-isopropylbenzene | https://webbook.nist.gov/cgi/cbook.cgi?ID=C535773 | benzene |
| Phenol, 4-ethyl- | https://webbook.nist.gov/cgi/cbook.cgi?ID=C123079 | benzene |
| Benzene, tert-butyl- | https://webbook.nist.gov/cgi/cbook.cgi?ID=C98066 | benzene |
| Benzene, 1,4-dichloro- | https://webbook.nist.gov/cgi/cbook.cgi?ID=C106467 | benzene |
| Benzene, (1-methylpropyl)- | https://webbook.nist.gov/cgi/cbook.cgi?ID=C135988 | benzene |
| Benzene, 1,2,3,5-tetramethyl- | https://webbook.nist.gov/cgi/cbook.cgi?ID=C527537 | benzene |
| Diethyl Phthalate | https://webbook.nist.gov/cgi/cbook.cgi?ID=C84662 | benzene |
| Ethanone, 1-(4-methylphenyl)- | https://webbook.nist.gov/cgi/cbook.cgi?ID=C122009 | benzene |
| Benzene, pentyl- | https://webbook.nist.gov/cgi/cbook.cgi?ID=C538681 | benzene |
| Benzene, 1,3-diethyl- | https://webbook.nist.gov/cgi/cbook.cgi?ID=C141935 | benzene |
| Benzophenone | https://webbook.nist.gov/cgi/cbook.cgi?ID=C119619 | benzene |
| Benzene, 1,2-dichloro- | https://webbook.nist.gov/cgi/cbook.cgi?ID=C95501 | benzene |
| 2,6-Di-t-butyl-p-cresol | https://webbook.nist.gov/cgi/cbook.cgi?ID=C128370 | benzene |
| 1-Methyl-3-propylbenzene | https://webbook.nist.gov/cgi/cbook.cgi?ID=C1074437 | benzene |
| Benzene, (2-methylpropyl)- | https://webbook.nist.gov/cgi/cbook.cgi?ID=C538932 | benzene |
| Benzene, 1,4-diethyl- | https://webbook.nist.gov/cgi/cbook.cgi?ID=C105055 | benzene |
| Benzene, 1,2,3,4-tetramethyl- | https://webbook.nist.gov/cgi/cbook.cgi?ID=C488233 | benzene |
| Benzene, nitro- | https://webbook.nist.gov/cgi/cbook.cgi?ID=C98953 | aniline |
| 1,3-Benzodioxole, 5-(2-propenyl)- | https://webbook.nist.gov/cgi/cbook.cgi?ID=C94597 | benzene |
| 1,2-Dimethyl-4-ethylbenzene | https://webbook.nist.gov/cgi/cbook.cgi?ID=C934805 | benzene |
| Benzonitrile | https://webbook.nist.gov/cgi/cbook.cgi?ID=C100470 | benzene |
| Benzene, bromo- | https://webbook.nist.gov/cgi/cbook.cgi?ID=C108861 | benzene |
| Phenol, 2,6-dimethyl- | https://webbook.nist.gov/cgi/cbook.cgi?ID=C576261 | benzene |
| Benzene, 1,3-dichloro- | https://webbook.nist.gov/cgi/cbook.cgi?ID=C541731 | benzene |
| 1-Methyl-4-propylbenzene | https://webbook.nist.gov/cgi/cbook.cgi?ID=C1074551 | benzene |
| Benzene, 1,2-diethyl- | https://webbook.nist.gov/cgi/cbook.cgi?ID=C135013 | benzene |
| .alpha.-Methylstyrene | https://webbook.nist.gov/cgi/cbook.cgi?ID=C98839 | benzene |
| Benzene, hexyl- | https://webbook.nist.gov/cgi/cbook.cgi?ID=C1077163 | benzene |
| Benzyl cyanide | https://webbook.nist.gov/cgi/cbook.cgi?ID=C140294 | benzene |
| Phenol, 3-ethyl- | https://webbook.nist.gov/cgi/cbook.cgi?ID=C620177 | benzene |
| Phenol, 2,4-dimethyl- | https://webbook.nist.gov/cgi/cbook.cgi?ID=C105679 | benzene |
| Benzene, 1,2-dimethoxy- | https://webbook.nist.gov/cgi/cbook.cgi?ID=C91167 | benzene |
| 1,4-Dimethyl-2-ethylbenzene | https://webbook.nist.gov/cgi/cbook.cgi?ID=C1758889 | benzene |
| Benzene, hexafluoro- | https://webbook.nist.gov/cgi/cbook.cgi?ID=C392563 | benzene |
| 1,3-Dimethyl-4-ethylbenzene | https://webbook.nist.gov/cgi/cbook.cgi?ID=C874419 | benzene |
| Pentamethylbenzene | https://webbook.nist.gov/cgi/cbook.cgi?ID=C700129 | benzene |
| Benzene, hexamethyl- | https://webbook.nist.gov/cgi/cbook.cgi?ID=C87854 | benzene |
| 1,2-Dimethyl-3-ethylbenzene | https://webbook.nist.gov/cgi/cbook.cgi?ID=C933982 | benzene |
| Isoeugenol | https://webbook.nist.gov/cgi/cbook.cgi?ID=C97541 | benzene |
| Phenol, 2-ethyl- | https://webbook.nist.gov/cgi/cbook.cgi?ID=C90006 | benzene |
| Benzene, fluoro- | https://webbook.nist.gov/cgi/cbook.cgi?ID=C462066 | benzene |
| Benzene, 1,2,4-trichloro- | https://webbook.nist.gov/cgi/cbook.cgi?ID=C120821 | benzene |
| 1,3-Cyclohexadiene | https://webbook.nist.gov/cgi/cbook.cgi?ID=C592574 | None |
| Phenol, 2,6-dimethoxy- | https://webbook.nist.gov/cgi/cbook.cgi?ID=C91101 | benzene |
| 1,4-Cyclohexadiene | https://webbook.nist.gov/cgi/cbook.cgi?ID=C628411 | None |
| Diphenyl ether | https://webbook.nist.gov/cgi/cbook.cgi?ID=C101848 | benzene |
| Benzene, hexachloro- | https://webbook.nist.gov/cgi/cbook.cgi?ID=C118741 | benzene |
| Benzenepropanol | https://webbook.nist.gov/cgi/cbook.cgi?ID=C122974 | benzene |
| Benzenemethanol, .alpha.-methyl- | https://webbook.nist.gov/cgi/cbook.cgi?ID=C98851 | benzene |
| p-Diphenylbenzene | https://webbook.nist.gov/cgi/cbook.cgi?ID=C92944 | benzene |
| Benzene, p-diisopropyl-, | https://webbook.nist.gov/cgi/cbook.cgi?ID=C100185 | benzene |
| Benzene, 1-methoxy-4-methyl- | https://webbook.nist.gov/cgi/cbook.cgi?ID=C104938 | benzene |
| 4-tert-Butyltoluene | https://webbook.nist.gov/cgi/cbook.cgi?ID=C98511 | benzene |
| Phenol, 2,4-dichloro- | https://webbook.nist.gov/cgi/cbook.cgi?ID=C120832 | benzene |
| Benzene, 1,2,4,5-tetrachloro- | https://webbook.nist.gov/cgi/cbook.cgi?ID=C95943 | benzene |
| Benzene, 1,3,5-trichloro- | https://webbook.nist.gov/cgi/cbook.cgi?ID=C108703 | benzene |
| 1,3-Dimethyl-2-ethylbenzene | https://webbook.nist.gov/cgi/cbook.cgi?ID=C2870044 | benzene |
| Hydroquinone | https://webbook.nist.gov/cgi/cbook.cgi?ID=C123319 | benzene |
| Benzene, 1,3,5-triethyl- | https://webbook.nist.gov/cgi/cbook.cgi?ID=C102250 | benzene |
| Benzene, octyl- | https://webbook.nist.gov/cgi/cbook.cgi?ID=C2189608 | benzene |
| Benzene, 1,2,3-trichloro- | https://webbook.nist.gov/cgi/cbook.cgi?ID=C87616 | benzene |
| Triphenylmethane | https://webbook.nist.gov/cgi/cbook.cgi?ID=C519733 | benzene |
| Ephedrine | https://webbook.nist.gov/cgi/cbook.cgi?ID=C299423 | benzene |
| 1,1'-Biphenyl, 4-methyl- | https://webbook.nist.gov/cgi/cbook.cgi?ID=C644086 | benzene |
| Phenylethyne | https://webbook.nist.gov/cgi/cbook.cgi?ID=C536743 | benzene |
| Resorcinol | https://webbook.nist.gov/cgi/cbook.cgi?ID=C108463 | benzene |
| Amphetamine | https://webbook.nist.gov/cgi/cbook.cgi?ID=C300629 | benzene |
| Phenol, 2-chloro- | https://webbook.nist.gov/cgi/cbook.cgi?ID=C95578 | benzene |
| Phenol, p-tert-butyl- | https://webbook.nist.gov/cgi/cbook.cgi?ID=C98544 | benzene |
| Lindane | https://webbook.nist.gov/cgi/cbook.cgi?ID=C58899 | None |
| Parachlorophenol | https://webbook.nist.gov/cgi/cbook.cgi?ID=C106489 | benzene |
| Catechol | https://webbook.nist.gov/cgi/cbook.cgi?ID=C120809 | benzene |
| Benzene, 1,4-dimethoxy- | https://webbook.nist.gov/cgi/cbook.cgi?ID=C150787 | benzene |
| Phenol, 2-methoxy-4-propyl- | https://webbook.nist.gov/cgi/cbook.cgi?ID=C2785877 | benzene |
| Phenol, 2,4,6-trimethyl- | https://webbook.nist.gov/cgi/cbook.cgi?ID=C527606 | benzene |
| Benzene, 1,3-bis(1-methylethyl)- | https://webbook.nist.gov/cgi/cbook.cgi?ID=C99627 | benzene |
| Benzene, pentachloro- | https://webbook.nist.gov/cgi/cbook.cgi?ID=C608935 | benzene |
| Benzene, 1,2,3,4-tetrachloro- | https://webbook.nist.gov/cgi/cbook.cgi?ID=C634662 | benzene |
| tert-Pentylbenzene | https://webbook.nist.gov/cgi/cbook.cgi?ID=C2049958 | benzene |
| 4,4'-Dimethylbiphenyl | https://webbook.nist.gov/cgi/cbook.cgi?ID=C613332 | benzene |
| Benzene, 2-propenyl- | https://webbook.nist.gov/cgi/cbook.cgi?ID=C300572 | benzene |
| Phenol, 2,4,6-trichloro- | https://webbook.nist.gov/cgi/cbook.cgi?ID=C88062 | benzene |
| Benzene, decyl- | https://webbook.nist.gov/cgi/cbook.cgi?ID=C104723 | benzene |
| Benzene, 1-chloro-4-methyl- | https://webbook.nist.gov/cgi/cbook.cgi?ID=C106434 | benzene |
| Benzene, 1,2,3,5-tetrachloro- | https://webbook.nist.gov/cgi/cbook.cgi?ID=C634902 | benzene |
| Benzene, 1-methyl-4-nitro- | https://webbook.nist.gov/cgi/cbook.cgi?ID=C99990 | aniline |
| Benzene, cyclohexyl- | https://webbook.nist.gov/cgi/cbook.cgi?ID=C827521 | benzene |
| Benzene, 1-chloro-2-methyl- | https://webbook.nist.gov/cgi/cbook.cgi?ID=C95498 | benzene |
| 1,4-Benzoquinone | https://webbook.nist.gov/cgi/cbook.cgi?ID=C106514 | None |
| Heptylbenzene | https://webbook.nist.gov/cgi/cbook.cgi?ID=C1078713 | benzene |
| o-Terphenyl | https://webbook.nist.gov/cgi/cbook.cgi?ID=C84151 | benzene |
| 1-Propanone, 1-phenyl- | https://webbook.nist.gov/cgi/cbook.cgi?ID=C93550 | benzene |
| Benzene, iodo- | https://webbook.nist.gov/cgi/cbook.cgi?ID=C591504 | benzene |
| Thiophenol | https://webbook.nist.gov/cgi/cbook.cgi?ID=C108985 | benzene |
| 3-Phenyl-1-propanol acetate | https://webbook.nist.gov/cgi/cbook.cgi?ID=C122725 | benzene |
| 2-Methylbiphenyl | https://webbook.nist.gov/cgi/cbook.cgi?ID=C643583 | benzene |
| Phenol, 3-chloro- | https://webbook.nist.gov/cgi/cbook.cgi?ID=C108430 | benzene |
| Phenol, 4-(1-methylethyl)- | https://webbook.nist.gov/cgi/cbook.cgi?ID=C99898 | benzene |
| 3-Methylstyrene | https://webbook.nist.gov/cgi/cbook.cgi?ID=C100801 | benzene |
| 4-Methylstyrene | https://webbook.nist.gov/cgi/cbook.cgi?ID=C622979 | benzene |
| 2,5-Dimethylstyrene | https://webbook.nist.gov/cgi/cbook.cgi?ID=C2039896 | benzene |
| Benzyl chloride | https://webbook.nist.gov/cgi/cbook.cgi?ID=C100447 | benzene |
| 3,3'-Dimethylbiphenyl | https://webbook.nist.gov/cgi/cbook.cgi?ID=C612759 | benzene |
| 4,4'-DDT | https://webbook.nist.gov/cgi/cbook.cgi?ID=C50293 | benzene |
| 1,3,5-Tri-isopropylbenzene | https://webbook.nist.gov/cgi/cbook.cgi?ID=C717748 | benzene |
| Benzene, 1-bromo-2-methyl- | https://webbook.nist.gov/cgi/cbook.cgi?ID=C95465 | benzene |
| 1,1'-Biphenyl, 3-methyl- | https://webbook.nist.gov/cgi/cbook.cgi?ID=C643936 | benzene |
| Phenol, 2-nitro- | https://webbook.nist.gov/cgi/cbook.cgi?ID=C88755 | aniline |
| Biphenylene | https://webbook.nist.gov/cgi/cbook.cgi?ID=C259790 | benzene |
| 4-Nitrophenol | https://webbook.nist.gov/cgi/cbook.cgi?ID=C100027 | aniline |
| Pentachlorophenol | https://webbook.nist.gov/cgi/cbook.cgi?ID=C87865 | benzene |
| 2-Methylstyrene | https://webbook.nist.gov/cgi/cbook.cgi?ID=C611154 | benzene |
| Phenol, 2,3,5-trimethyl- | https://webbook.nist.gov/cgi/cbook.cgi?ID=C697825 | benzene |
| Benzene, 1-methyl-2,4-dinitro- | https://webbook.nist.gov/cgi/cbook.cgi?ID=C121142 | aniline |
| Phenol, 2-(1-methylethyl)- | https://webbook.nist.gov/cgi/cbook.cgi?ID=C88697 | benzene |
| Benzene, 1-methoxy-3-methyl- | https://webbook.nist.gov/cgi/cbook.cgi?ID=C100845 | benzene |
| (3-Methylbutyl)benzene | https://webbook.nist.gov/cgi/cbook.cgi?ID=C2049947 | benzene |
| Benzene, (trifluoromethyl)- | https://webbook.nist.gov/cgi/cbook.cgi?ID=C98088 | benzene |
| Benzylamine | https://webbook.nist.gov/cgi/cbook.cgi?ID=C100469 | benzene |
| Pentafluorobenzene | https://webbook.nist.gov/cgi/cbook.cgi?ID=C363724 | benzene |
| Benzene, 1-chloro-3-methyl- | https://webbook.nist.gov/cgi/cbook.cgi?ID=C108418 | benzene |
| Nonylbenzene | https://webbook.nist.gov/cgi/cbook.cgi?ID=C1081772 | benzene |
| Benzene, 1,3,5-trichloro-2-methoxy- | https://webbook.nist.gov/cgi/cbook.cgi?ID=C87401 | benzene |
| o-Methylanisole | https://webbook.nist.gov/cgi/cbook.cgi?ID=C578585 | benzene |
| Diphenylacetylene | https://webbook.nist.gov/cgi/cbook.cgi?ID=C501655 | benzene |
| 1,3,5-Triphenylbenzene | https://webbook.nist.gov/cgi/cbook.cgi?ID=C612715 | benzene |
| 1-Ethyl-4-isopropylbenzene | https://webbook.nist.gov/cgi/cbook.cgi?ID=C4218488 | benzene |
| Benzene, 1,4-dibromo- | https://webbook.nist.gov/cgi/cbook.cgi?ID=C106376 | benzene |
| Benzene, 2-methyl-1,3-dinitro- | https://webbook.nist.gov/cgi/cbook.cgi?ID=C606202 | aniline |
| Benzene, 1-chloro-4-nitro- | https://webbook.nist.gov/cgi/cbook.cgi?ID=C100005 | aniline |
| 1,3-Dinitrobenzene | https://webbook.nist.gov/cgi/cbook.cgi?ID=C99650 | aniline |
| 1-Ethyl-3-isopropylbenzene | https://webbook.nist.gov/cgi/cbook.cgi?ID=C4920994 | benzene |
| Benzene, 1-methyl-2-nitro- | https://webbook.nist.gov/cgi/cbook.cgi?ID=C88722 | aniline |
| 4,4'-DDE | https://webbook.nist.gov/cgi/cbook.cgi?ID=C72559 | benzene |
| 1-tert-Butyl-3-methylbenzene | https://webbook.nist.gov/cgi/cbook.cgi?ID=C1075383 | benzene |
| Benzene, 1,2-dimethoxy-4-(1-propenyl)- | https://webbook.nist.gov/cgi/cbook.cgi?ID=C93163 | benzene |
| 1,3-Diazine | https://webbook.nist.gov/cgi/cbook.cgi?ID=C289952 | None |
| 1,2,4-Triethylbenzene | https://webbook.nist.gov/cgi/cbook.cgi?ID=C877441 | benzene |
| 1,3-Diethyl-5-methylbenzene | https://webbook.nist.gov/cgi/cbook.cgi?ID=C2050240 | benzene |
| o-Hydroxybiphenyl | https://webbook.nist.gov/cgi/cbook.cgi?ID=C90437 | benzene |
| Benzene, 1-methoxy-4-(1-propenyl)- | https://webbook.nist.gov/cgi/cbook.cgi?ID=C104461 | benzene |
| Benzene, 1-bromo-4-methyl- | https://webbook.nist.gov/cgi/cbook.cgi?ID=C106387 | benzene |
| Phenol, 2,3,4,6-tetrachloro- | https://webbook.nist.gov/cgi/cbook.cgi?ID=C58902 | benzene |
| Benzene, cyclopropyl- | https://webbook.nist.gov/cgi/cbook.cgi?ID=C873494 | benzene |
| Phenylsulfide | https://webbook.nist.gov/cgi/cbook.cgi?ID=C139662 | benzene |
| Benzene, 1-(1,1-dimethylethyl)-3,5-dimethyl- | https://webbook.nist.gov/cgi/cbook.cgi?ID=C98191 | benzene |
| (1-Methybutyl)benzene | https://webbook.nist.gov/cgi/cbook.cgi?ID=C2719520 | benzene |
| p-n-Propylphenol | https://webbook.nist.gov/cgi/cbook.cgi?ID=C645567 | benzene |
| 1,3-Dimethoxybenzene | https://webbook.nist.gov/cgi/cbook.cgi?ID=C151100 | benzene |
| Phenol, 2-propyl- | https://webbook.nist.gov/cgi/cbook.cgi?ID=C644359 | benzene |
| Benzene, 1-chloro-4-methoxy- | https://webbook.nist.gov/cgi/cbook.cgi?ID=C623121 | benzene |
| Phenol, 3-nitro- | https://webbook.nist.gov/cgi/cbook.cgi?ID=C554847 | aniline |
| Phenethylamine | https://webbook.nist.gov/cgi/cbook.cgi?ID=C64040 | benzene |
| Benzyl methyl sulfide | https://webbook.nist.gov/cgi/cbook.cgi?ID=C766927 | benzene |
| Benzene, 1,2-difluoro- | https://webbook.nist.gov/cgi/cbook.cgi?ID=C367113 | benzene |
| Phenol, 4-(1-methylpropyl)- | https://webbook.nist.gov/cgi/cbook.cgi?ID=C99718 | benzene |
| cis-Propenylbenzene | https://webbook.nist.gov/cgi/cbook.cgi?ID=C873665 | benzene |
| Benzenemethanethiol | https://webbook.nist.gov/cgi/cbook.cgi?ID=C100538 | benzene |
| Benzene, 1-chloro-3-nitro- | https://webbook.nist.gov/cgi/cbook.cgi?ID=C121733 | aniline |
| Benzene, 1,2-dimethoxy-4-methyl- | https://webbook.nist.gov/cgi/cbook.cgi?ID=C494995 | benzene |
| 4-Vinylanisole | https://webbook.nist.gov/cgi/cbook.cgi?ID=C637694 | benzene |
| Benzene, 1-bromo-3-methyl- | https://webbook.nist.gov/cgi/cbook.cgi?ID=C591173 | benzene |
| Benzene, 1-bromo-2-chloro- | https://webbook.nist.gov/cgi/cbook.cgi?ID=C694804 | benzene |
| 4-Chloro-3-methylphenol | https://webbook.nist.gov/cgi/cbook.cgi?ID=C59507 | benzene |
| Benzene, 1-chloro-2-nitro- | https://webbook.nist.gov/cgi/cbook.cgi?ID=C88733 | aniline |
| 1,1'-Biphenyl, 4-chloro- | https://webbook.nist.gov/cgi/cbook.cgi?ID=C2051629 | benzene |
| Benzene, pentafluoromethyl- | https://webbook.nist.gov/cgi/cbook.cgi?ID=C771562 | benzene |
| Benzene, 1,4-difluoro- | https://webbook.nist.gov/cgi/cbook.cgi?ID=C540363 | benzene |
| Benzene, 1-methyl-3-nitro- | https://webbook.nist.gov/cgi/cbook.cgi?ID=C99081 | aniline |
| Mequinol | https://webbook.nist.gov/cgi/cbook.cgi?ID=C150765 | benzene |
| Benzene, pentafluoro(trifluoromethyl)- | https://webbook.nist.gov/cgi/cbook.cgi?ID=C434640 | benzene |
| Benzene, 1-chloro-2-methoxy- | https://webbook.nist.gov/cgi/cbook.cgi?ID=C766518 | benzene |
| 1,4-Dinitrobenzene | https://webbook.nist.gov/cgi/cbook.cgi?ID=C100254 | aniline |
| o,p'-DDE | https://webbook.nist.gov/cgi/cbook.cgi?ID=C3424826 | benzene |
| Benzene, (methoxymethyl)- | https://webbook.nist.gov/cgi/cbook.cgi?ID=C538863 | benzene |
| Benzene, 1,3-difluoro- | https://webbook.nist.gov/cgi/cbook.cgi?ID=C372189 | benzene |
| Benzene, 1,3-dibromo- | https://webbook.nist.gov/cgi/cbook.cgi?ID=C108361 | benzene |
| 1,3-Diethyl-4-methylbenzene | https://webbook.nist.gov/cgi/cbook.cgi?ID=C1758856 | benzene |
| 1,2,3,4-Tetrafluorobenzene | https://webbook.nist.gov/cgi/cbook.cgi?ID=C551622 | benzene |
| 3-Chloroanisole | https://webbook.nist.gov/cgi/cbook.cgi?ID=C2845898 | benzene |
| Oxirane, (phenoxymethyl)- | https://webbook.nist.gov/cgi/cbook.cgi?ID=C122601 | benzene |
| Benzene, 1,3-dimethyl-5-(1-methylethyl)- | https://webbook.nist.gov/cgi/cbook.cgi?ID=C4706905 | benzene |
| Benzene, 1,2,4-trichloro-3-methoxy- | https://webbook.nist.gov/cgi/cbook.cgi?ID=C50375105 | benzene |
| p-Hydroxybiphenyl | https://webbook.nist.gov/cgi/cbook.cgi?ID=C92693 | benzene |
| Benzene, 1,2-dimethyl-4-(1-methylethyl)- | https://webbook.nist.gov/cgi/cbook.cgi?ID=C4132778 | benzene |
| Benzene, 1,4-diiodo- | https://webbook.nist.gov/cgi/cbook.cgi?ID=C624384 | benzene |
| Benzene, 1-fluoro-3-methyl- | https://webbook.nist.gov/cgi/cbook.cgi?ID=C352705 | benzene |
| Phenol, 2-(1,1-dimethylethyl)-4-methyl- | https://webbook.nist.gov/cgi/cbook.cgi?ID=C2409554 | benzene |
| Benzene, 1,2-dibromo- | https://webbook.nist.gov/cgi/cbook.cgi?ID=C583539 | benzene |
| 1,4-Diethyl-2-methylbenzene | https://webbook.nist.gov/cgi/cbook.cgi?ID=C13632945 | benzene |
| Benzylbromide | https://webbook.nist.gov/cgi/cbook.cgi?ID=C100390 | benzene |
| .alpha.-Chloroacetophenone | https://webbook.nist.gov/cgi/cbook.cgi?ID=C532274 | benzene |
| Ethanone, 1-(4-ethylphenyl)- | https://webbook.nist.gov/cgi/cbook.cgi?ID=C937304 | benzene |
| Neopentylbenzene | https://webbook.nist.gov/cgi/cbook.cgi?ID=C1007267 | benzene |
| Pyrogallol | https://webbook.nist.gov/cgi/cbook.cgi?ID=C87661 | benzene |
| PCP, Methyl ether | https://webbook.nist.gov/cgi/cbook.cgi?ID=C1825214 | benzene |
| Benzene, pentachloronitro- | https://webbook.nist.gov/cgi/cbook.cgi?ID=C82688 | aniline |
| 2-Methyl-1-phenyl-1-propene | https://webbook.nist.gov/cgi/cbook.cgi?ID=C768490 | benzene |
| 1,1'-Biphenyl, 4-nitro- | https://webbook.nist.gov/cgi/cbook.cgi?ID=C92933 | simult_aniline&benzene |
| Phenol, 4,4'-(1-methylethylidene)bis- | https://webbook.nist.gov/cgi/cbook.cgi?ID=C80057 | benzene |
| Benzene, 1,2-dichloro-3-methoxy- | https://webbook.nist.gov/cgi/cbook.cgi?ID=C1984594 | benzene |
| Benzene, 2,4-dichloro-1-methoxy- | https://webbook.nist.gov/cgi/cbook.cgi?ID=C553822 | benzene |
| Phenol, 2,4-dinitro- | https://webbook.nist.gov/cgi/cbook.cgi?ID=C51285 | aniline |
| Azobenzene | https://webbook.nist.gov/cgi/cbook.cgi?ID=C103333 | aniline |
| p-Bromobenzoic acid | https://webbook.nist.gov/cgi/cbook.cgi?ID=C586765 | aniline |
| (2-Methylbutyl)benzene | https://webbook.nist.gov/cgi/cbook.cgi?ID=C3968852 | benzene |
| Benzene, dodecyl- | https://webbook.nist.gov/cgi/cbook.cgi?ID=C123013 | benzene |
| Hydrocinnamonitrile | https://webbook.nist.gov/cgi/cbook.cgi?ID=C645590 | benzene |
| Methoxychlor | https://webbook.nist.gov/cgi/cbook.cgi?ID=C72435 | benzene |
| Benzene, (ethoxymethyl)- | https://webbook.nist.gov/cgi/cbook.cgi?ID=C539300 | benzene |
| 1,2-Dinitrobenzene | https://webbook.nist.gov/cgi/cbook.cgi?ID=C528290 | aniline |
| (1-Ethylpropyl)benzene | https://webbook.nist.gov/cgi/cbook.cgi?ID=C1196583 | benzene |
| 1-t-Butyl-4-ethylbenzene | https://webbook.nist.gov/cgi/cbook.cgi?ID=C7364194 | benzene |
| 1,2,4,5-Tetrafluorobenzene | https://webbook.nist.gov/cgi/cbook.cgi?ID=C327548 | benzene |
| Benzene, 1-bromo-4-chloro- | https://webbook.nist.gov/cgi/cbook.cgi?ID=C106398 | benzene |
| 1-tert-Butyl-2-methylbenzene | https://webbook.nist.gov/cgi/cbook.cgi?ID=C1074926 | benzene |
| 1-Chloro-4-ethylbenzene | https://webbook.nist.gov/cgi/cbook.cgi?ID=C622980 | benzene |
| p-Bromoanisole | https://webbook.nist.gov/cgi/cbook.cgi?ID=C104927 | benzene |
| Benzoic acid, 3,4-dimethyl- | https://webbook.nist.gov/cgi/cbook.cgi?ID=C619045 | benzene |
| Benzene, 1-iodo-4-methyl- | https://webbook.nist.gov/cgi/cbook.cgi?ID=C624317 | benzene |
| 3'-Methoxyacetophenone | https://webbook.nist.gov/cgi/cbook.cgi?ID=C586378 | benzene |
| Benzene, isocyanato- | https://webbook.nist.gov/cgi/cbook.cgi?ID=C103719 | aniline |
| Benzonitrile, 4-methyl- | https://webbook.nist.gov/cgi/cbook.cgi?ID=C104858 | benzene |
| Phenol, 3-ethyl-5-methyl- | https://webbook.nist.gov/cgi/cbook.cgi?ID=C698715 | benzene |
| o-Chloroethylbenzene | https://webbook.nist.gov/cgi/cbook.cgi?ID=C89963 | benzene |
| Benzene, 4-ethenyl-1,2-dimethoxy- | https://webbook.nist.gov/cgi/cbook.cgi?ID=C6380230 | benzene |
| Benzyl fluoride | https://webbook.nist.gov/cgi/cbook.cgi?ID=C350505 | benzene |
| Benzoic acid, 4-nitro- | https://webbook.nist.gov/cgi/cbook.cgi?ID=C62237 | aniline |
| Benzene, 1-bromo-4-phenoxy- | https://webbook.nist.gov/cgi/cbook.cgi?ID=C101553 | benzene |
| 1,3-Ditertiarybutylbenzene | https://webbook.nist.gov/cgi/cbook.cgi?ID=C1014604 | benzene |
| 4-Methyldiphenylmethane | https://webbook.nist.gov/cgi/cbook.cgi?ID=C620837 | benzene |
| Benzene, 2-butenyl- | https://webbook.nist.gov/cgi/cbook.cgi?ID=C1560061 | benzene |
| Benzenemethanol, 4-methyl- | https://webbook.nist.gov/cgi/cbook.cgi?ID=C589184 | benzene |
| 2,4,6-Tri-tert-butylphenol | https://webbook.nist.gov/cgi/cbook.cgi?ID=C732263 | benzene |
| Phenol, 2,4,6-tribromo- | https://webbook.nist.gov/cgi/cbook.cgi?ID=C118796 | benzene |
| 5-Isopropyl-m-xylene | https://webbook.nist.gov/cgi/cbook.cgi?ID=C4706892 | benzene |
| Benzene, (2-chloroethyl)- | https://webbook.nist.gov/cgi/cbook.cgi?ID=C622242 | benzene |
| 4-Methylcatechol | https://webbook.nist.gov/cgi/cbook.cgi?ID=C452868 | benzene |
| Benzene, (trichloromethyl)- | https://webbook.nist.gov/cgi/cbook.cgi?ID=C98077 | benzene |
| Benzene, 1,3,5-trimethyl-2-nitro- | https://webbook.nist.gov/cgi/cbook.cgi?ID=C603714 | aniline |
| Benzyl sulfide | https://webbook.nist.gov/cgi/cbook.cgi?ID=C538749 | benzene |
| Benzene, 1-methoxy-4-nitro- | https://webbook.nist.gov/cgi/cbook.cgi?ID=C100174 | aniline |
| Benzene, propoxy- | https://webbook.nist.gov/cgi/cbook.cgi?ID=C622855 | benzene |
| Phenol, 2,4,5-trimethyl- | https://webbook.nist.gov/cgi/cbook.cgi?ID=C496786 | benzene |
| Benzene, 1-propynyl- | https://webbook.nist.gov/cgi/cbook.cgi?ID=C673325 | benzene |
| Octadecylbenzene | https://webbook.nist.gov/cgi/cbook.cgi?ID=C4445072 | benzene |
| Benzotriazole | https://webbook.nist.gov/cgi/cbook.cgi?ID=C95147 | benzene |
| 4-Fluoroanisole | https://webbook.nist.gov/cgi/cbook.cgi?ID=C459609 | benzene |
| Benzene, 3-butenyl- | https://webbook.nist.gov/cgi/cbook.cgi?ID=C768569 | benzene |
| Ethanone, 2,2,2-trifluoro-1-phenyl- | https://webbook.nist.gov/cgi/cbook.cgi?ID=C434457 | benzene |
| Acetophenone, 4'-nitro- | https://webbook.nist.gov/cgi/cbook.cgi?ID=C100196 | aniline |
| 1-Allyl-2-methylbenzene | https://webbook.nist.gov/cgi/cbook.cgi?ID=C1587048 | benzene |
| Benzene, 1-chloro-4-fluoro- | https://webbook.nist.gov/cgi/cbook.cgi?ID=C352330 | benzene |
| Piperidine | https://webbook.nist.gov/cgi/cbook.cgi?ID=C110894 | piperidine |
| 2-Methylpiperidine | https://webbook.nist.gov/cgi/cbook.cgi?ID=C109057 | piperidine |
| Piperidine, 1-ethyl- | https://webbook.nist.gov/cgi/cbook.cgi?ID=C766096 | piperidine |
| Piperidine, 4-methyl- | https://webbook.nist.gov/cgi/cbook.cgi?ID=C626584 | piperidine |
| Nanofin | https://webbook.nist.gov/cgi/cbook.cgi?ID=C504030 | piperidine |
| 2-Ethylpiperidine | https://webbook.nist.gov/cgi/cbook.cgi?ID=C1484806 | piperidine |
| 4-Benzylpiperidine | https://webbook.nist.gov/cgi/cbook.cgi?ID=C31252423 | piperidine |
| 1-Methyl-2-piperidinemethanol | https://webbook.nist.gov/cgi/cbook.cgi?ID=C20845345 | piperidine |
| 1-Benzyl-4-piperidine | https://webbook.nist.gov/cgi/cbook.cgi?ID=C3612202 | piperidine |
| 4-(Aminomethyl)piperidine | https://webbook.nist.gov/cgi/cbook.cgi?ID=C7144050 | piperidine |
| 1,3-Di-(4-piperidyl)propane | https://webbook.nist.gov/cgi/cbook.cgi?ID=C16898525 | piperidine |
| Aniline | https://webbook.nist.gov/cgi/cbook.cgi?ID=C62533 | aniline |
| Ethanone, 1-(2-aminophenyl)- | https://webbook.nist.gov/cgi/cbook.cgi?ID=C551939 | aniline |
| Benzenamine, N,N-dimethyl- | https://webbook.nist.gov/cgi/cbook.cgi?ID=C121697 | aniline |
| Benzenamine, 2-methyl- | https://webbook.nist.gov/cgi/cbook.cgi?ID=C95534 | aniline |
| Diphenylamine | https://webbook.nist.gov/cgi/cbook.cgi?ID=C122394 | aniline |
| p-Aminotoluene | https://webbook.nist.gov/cgi/cbook.cgi?ID=C106490 | aniline |
| Benzenamine, 3-methyl- | https://webbook.nist.gov/cgi/cbook.cgi?ID=C108441 | aniline |
| Aniline, N-methyl- | https://webbook.nist.gov/cgi/cbook.cgi?ID=C100618 | aniline |
| Cyclohexanamine | https://webbook.nist.gov/cgi/cbook.cgi?ID=C108918 | None |
| p-Chloroaniline | https://webbook.nist.gov/cgi/cbook.cgi?ID=C106478 | aniline |
| 3-Nitroaniline | https://webbook.nist.gov/cgi/cbook.cgi?ID=C99092 | aniline |
| m-Chloroaniline | https://webbook.nist.gov/cgi/cbook.cgi?ID=C108429 | aniline |
| o-Chloroaniline | https://webbook.nist.gov/cgi/cbook.cgi?ID=C95512 | aniline |
| Benzenamine, N-ethyl- | https://webbook.nist.gov/cgi/cbook.cgi?ID=C103695 | aniline |
| Benzenamine, N,N-diethyl- | https://webbook.nist.gov/cgi/cbook.cgi?ID=C91667 | aniline |
| Phenol, 4-amino- | https://webbook.nist.gov/cgi/cbook.cgi?ID=C123308 | aniline |
| Trifluralin | https://webbook.nist.gov/cgi/cbook.cgi?ID=C1582098 | aniline |
| Benzenamine, 2,4-dimethyl- | https://webbook.nist.gov/cgi/cbook.cgi?ID=C95681 | aniline |
| 2-Nitroaniline | https://webbook.nist.gov/cgi/cbook.cgi?ID=C88744 | aniline |
| p-Aminobenzoic acid | https://webbook.nist.gov/cgi/cbook.cgi?ID=C150130 | aniline |
| 2-Aminobenzoic acid | https://webbook.nist.gov/cgi/cbook.cgi?ID=C118923 | aniline |
| Benzenamine, N,N,4-trimethyl- | https://webbook.nist.gov/cgi/cbook.cgi?ID=C99978 | aniline |
| 1,4-Benzenediamine | https://webbook.nist.gov/cgi/cbook.cgi?ID=C106503 | aniline |
| 1,3-Benzenediamine | https://webbook.nist.gov/cgi/cbook.cgi?ID=C108452 | aniline |
| Phenol, 3-amino- | https://webbook.nist.gov/cgi/cbook.cgi?ID=C591275 | aniline |
| p-Anisidine | https://webbook.nist.gov/cgi/cbook.cgi?ID=C104949 | aniline |
| 1,2-Benzenediamine | https://webbook.nist.gov/cgi/cbook.cgi?ID=C95545 | aniline |
| Benzenamine, 3,4-dichloro- | https://webbook.nist.gov/cgi/cbook.cgi?ID=C95761 | aniline |
| Phenol, 2-amino- | https://webbook.nist.gov/cgi/cbook.cgi?ID=C95556 | aniline |
| Acetophenone, 4'-amino- | https://webbook.nist.gov/cgi/cbook.cgi?ID=C99923 | aniline |
| 4-Ethylaniline | https://webbook.nist.gov/cgi/cbook.cgi?ID=C589162 | aniline |
| Benzenamine, 2,4,6-trichloro- | https://webbook.nist.gov/cgi/cbook.cgi?ID=C634935 | aniline |
| Botran | https://webbook.nist.gov/cgi/cbook.cgi?ID=C99309 | aniline |
| Benzenamine, 2,5-dichloro- | https://webbook.nist.gov/cgi/cbook.cgi?ID=C95829 | aniline |
| p-Fluoroaniline | https://webbook.nist.gov/cgi/cbook.cgi?ID=C371404 | aniline |
| Benzenamine, 3-bromo- | https://webbook.nist.gov/cgi/cbook.cgi?ID=C591195 | aniline |
| 1-Piperidinepropanol | https://webbook.nist.gov/cgi/cbook.cgi?ID=C104585&Units=SI | piperidine |
| Acetone | https://webbook.nist.gov/cgi/cbook.cgi?ID=C67641&Units=SI&Type=IR-SPEC&Index=2#IR-SPEC | None |
| Ethane | https://webbook.nist.gov/cgi/cbook.cgi?ID=C74840&Units=SI&Type=IR-SPEC&Index=1#IR-SPEC | None |
| Decane | https://webbook.nist.gov/cgi/cbook.cgi?Name=decane&Units=SI&cTG=on&cIR=on | None |
| Cyclobutanone | https://webbook.nist.gov/cgi/cbook.cgi?ID=C1191953&Units=SI&Type=IR-SPEC&Index=1#IR-SPEC | None |
| 2-Pentene, 2,4,4-trimethyl- | https://webbook.nist.gov/cgi/cbook.cgi?ID=C107404&Units=SI&Mask=81#IR-Spec | None |
| Phencylidine | https://webbook.nist.gov/cgi/cbook.cgi?ID=C77101 | piperidine |
| CYPROHEPTADINE | https://webbook.nist.gov/cgi/cbook.cgi?ID=C129033 | piperidine |
| 1,2,5,6-Tetrahydropyridine | https://webbook.nist.gov/cgi/cbook.cgi?ID=C694053 | piperidine |
| 2,2,6,6-Tetramethyl-4-piperidone | https://webbook.nist.gov/cgi/cbook.cgi?ID=C826368 | piperidine |
| 1,3-Dimethylpiperidine | https://webbook.nist.gov/cgi/cbook.cgi?ID=C695352 | piperidine |
| 4-PIPERIDINOL, 2,2,6,6-TETRAMETHYL-, 1-OXIDE | https://webbook.nist.gov/cgi/cbook.cgi?ID=C3637103 | piperidine |
| N-Cyanomethylpiperidine | https://webbook.nist.gov/cgi/cbook.cgi?ID=C3010035 | piperidine |
| 4-Hydroxy-1-methylpiperidine | https://webbook.nist.gov/cgi/cbook.cgi?ID=C106525 | piperidine |
| 4-Phenylpiperidine | https://webbook.nist.gov/cgi/cbook.cgi?ID=C771993 | piperidine |
| Methylendipiperidine | https://webbook.nist.gov/cgi/cbook.cgi?ID=C880091 | piperidine |
| 4-Piperidinol, 1-(phenylmethyl)- | https://webbook.nist.gov/cgi/cbook.cgi?ID=C4727724 | piperidine |
| N-Ethyl-4-hydroxypiperidine | https://webbook.nist.gov/cgi/cbook.cgi?ID=C3518830 | piperidine |
| 4-Hydroxypiperidine | https://webbook.nist.gov/cgi/cbook.cgi?ID=C5382161 | piperidine |
| Piperidineacetaldehyde diethyl acetal | https://webbook.nist.gov/cgi/cbook.cgi?ID=C3616588 | piperidine |
| 4-Piperidinepropanol, 1-(2-hydroxyethyl)- | https://webbook.nist.gov/cgi/cbook.cgi?ID=C19780859 | piperidine |
| 4-(4-Chlorophenyl)-4-hydroxypiperidine | https://webbook.nist.gov/cgi/cbook.cgi?ID=C39512497 | piperidine |
| 4-Benzyl-4-hydroxypiperidine | https://webbook.nist.gov/cgi/cbook.cgi?ID=C51135967 | piperidine |
| 4-Acetyl-4-phenylpiperidine hydrochloride | https://webbook.nist.gov/cgi/cbook.cgi?ID=C34798806 | piperidine |
| 2-n-Propylpiperidine | https://webbook.nist.gov/cgi/cbook.cgi?ID=C3238606 | piperidine |
| Piperidine, 4-(diphenylmethyl)- | https://webbook.nist.gov/cgi/cbook.cgi?ID=C19841737 | piperidine |
| 2,4,6-Trimethylpiperidine | https://webbook.nist.gov/cgi/cbook.cgi?ID=C21974481 | piperidine |
| Benzeneacetamide | https://webbook.nist.gov/cgi/cbook.cgi?ID=C103811&Units=SI | simult_Amide&benzene |
| Isophthalamide | https://webbook.nist.gov/cgi/cbook.cgi?ID=C1740574&Units=SI | simult_Amide&benzene |
| p-Dimethylaminoacetophenone | https://webbook.nist.gov/cgi/cbook.cgi?ID=C18925694&Units=SI | simult_Amide&benzene |
| 2-Methylaminobenzonitrile | https://webbook.nist.gov/cgi/cbook.cgi?ID=C7505819&Units=SI | simult_Amide&benzene |
| Phthalamide | https://webbook.nist.gov/cgi/cbook.cgi?ID=C88960&Units=SI | simult_Amide&benzene |
| GLUTETHIMIDE | https://webbook.nist.gov/cgi/cbook.cgi?ID=C77214&Units=SI&Mask=80 | simult_Amide&benzene |
| Benzeneacetamide | https://webbook.nist.gov/cgi/cbook.cgi?ID=C103811&Units=SI&Mask=80 | simult_Amide&benzene |
| Acetamide, N-phenethyl- | https://webbook.nist.gov/cgi/cbook.cgi?ID=C877952&Units=SI&Mask=80 | simult_Amide&benzene |
| DIBUCAINE | https://webbook.nist.gov/cgi/cbook.cgi?ID=C85790&Units=SI&Mask=80 | simult_Amide&benzene |
| Napropamide | https://webbook.nist.gov/cgi/cbook.cgi?ID=C15299997&Units=SI&Mask=80 | simult_Amide&benzene |
| ACETANILIDE, p-ETHOXY- | https://webbook.nist.gov/cgi/cbook.cgi?ID=C62442&Units=SI | simult_Amide&aniline |
| Acetamide, N-phenyl- | https://webbook.nist.gov/cgi/cbook.cgi?ID=C103844&Units=SI | simult_Amide&aniline |
| 4-Acetamidophenol | https://webbook.nist.gov/cgi/cbook.cgi?ID=C103902&Units=SI | simult_Amide&aniline |
| p-ACETANISIDIDE | https://webbook.nist.gov/cgi/cbook.cgi?ID=C51661&Units=SI | simult_Amide&aniline |
| FORMANILIDE | https://webbook.nist.gov/cgi/cbook.cgi?ID=C103708&Units=SI | simult_Amide&aniline |
| N-Phenylurethane | https://webbook.nist.gov/cgi/cbook.cgi?ID=C101995&Units=SI | simult_Amide&aniline |
| Formamide, N-methyl-N-phenyl- | https://webbook.nist.gov/cgi/cbook.cgi?ID=C93618&Units=SI | simult_Amide&aniline |
| N-2'-Chloroacetyl-2,6-diethylaniline | https://webbook.nist.gov/cgi/cbook.cgi?ID=C6967299&Units=SI | simult_Amide&aniline |
| ACETANILIDE, N-METHYL- | https://webbook.nist.gov/cgi/cbook.cgi?ID=C579102&Units=SI | simult_Amide&aniline |
| 4'-Bromoacetanilide | https://webbook.nist.gov/cgi/cbook.cgi?ID=C103888&Units=SI | simult_Amide&aniline |
| Metolachlor | https://webbook.nist.gov/cgi/cbook.cgi?ID=C51218452&Units=SI | simult_Amide&aniline |
| Acetamide, N-(2,6-dimethylphenyl)- | https://webbook.nist.gov/cgi/cbook.cgi?ID=C2198530&Units=SI | simult_Amide&aniline |
| ACETANILIDE, p-ETHOXY- | https://webbook.nist.gov/cgi/cbook.cgi?ID=C62442&Units=SI&Mask=80 | simult_Amide&aniline |
| Acetamide, N-phenyl- | https://webbook.nist.gov/cgi/cbook.cgi?ID=C103844&Units=SI&Mask=80 | simult_Amide&aniline |
| 4-Acetamidophenol | https://webbook.nist.gov/cgi/cbook.cgi?ID=C103902&Units=SI&Mask=80 | simult_Amide&aniline |
| Alachlor | https://webbook.nist.gov/cgi/cbook.cgi?ID=C15972608&Units=SI&Mask=80 | simult_Amide&aniline |
| Mepivacaine | https://webbook.nist.gov/cgi/cbook.cgi?ID=C96888&Units=SI&Mask=80 | simult_Amide&aniline |
| Propanil | https://webbook.nist.gov/cgi/cbook.cgi?ID=C709988&Units=SI&Mask=80 | simult_Amide&aniline |
| p-Ethoxybenzylidene p-butylaniline | https://webbook.nist.gov/cgi/cbook.cgi?ID=C29743086&Units=SI | simult_aniline&benzene |
| p-Hexyloxybenzylidene p-butylaniline | https://webbook.nist.gov/cgi/cbook.cgi?ID=C29743111&Units=SI | simult_aniline&benzene |
| 2-Phenoxyaniline | https://webbook.nist.gov/cgi/cbook.cgi?ID=C2688848&Units=SI | simult_aniline&benzene |
| N-(4-Methoxybenzylidene)aniline | https://webbook.nist.gov/cgi/cbook.cgi?ID=C836419&Units=SI | simult_aniline&benzene |
| Salicylidene aniline | https://webbook.nist.gov/cgi/cbook.cgi?ID=C779840&Units=SI | simult_aniline&benzene |
| p-Butoxybenzylidene p-butylaniline | https://webbook.nist.gov/cgi/cbook.cgi?ID=C29743097&Units=SI | simult_aniline&benzene |
| Methane, nitro- | https://webbook.nist.gov/cgi/cbook.cgi?ID=C75525 | None |
| 2-Propenenitrile | https://webbook.nist.gov/cgi/cbook.cgi?ID=C107131 | None |
| Ethane, nitro- | https://webbook.nist.gov/cgi/cbook.cgi?ID=C79243 | None |
| Ammonia | https://webbook.nist.gov/cgi/cbook.cgi?ID=C7664417 | None |
| Nitrous Oxide | https://webbook.nist.gov/cgi/cbook.cgi?ID=C10024972 | None |
| 1-Nitrobutane | https://webbook.nist.gov/cgi/cbook.cgi?ID=C627054 | None |
| Propane, 2-nitro- | https://webbook.nist.gov/cgi/cbook.cgi?ID=C79469 | None |
| ETHYL NITRATE | https://webbook.nist.gov/cgi/cbook.cgi?ID=C625581 | None |
| 3-Cyanopyridine | https://webbook.nist.gov/cgi/cbook.cgi?ID=C100549 | None |
| n-Decanenitrile | https://webbook.nist.gov/cgi/cbook.cgi?ID=C1975786 | None |
| n-Butyl nitrite | https://webbook.nist.gov/cgi/cbook.cgi?ID=C544161 | None |
| PENTAERYTHRITYL TETRANITRATE | https://webbook.nist.gov/cgi/cbook.cgi?ID=C78115 | None |
| Hexanedinitrile | https://webbook.nist.gov/cgi/cbook.cgi?ID=C111693 | None |
| TRIETHANOLAMINE | https://webbook.nist.gov/cgi/cbook.cgi?ID=C102716 | None |
| Propane, 2-methyl-2-nitro- | https://webbook.nist.gov/cgi/cbook.cgi?ID=C594707 | None |
| Propyl nitrate | https://webbook.nist.gov/cgi/cbook.cgi?ID=C627134 | None |
| N-Nitrosodimethylamine | https://webbook.nist.gov/cgi/cbook.cgi?ID=C62759 | None |
| Diethylaminoacetonitrile | https://webbook.nist.gov/cgi/cbook.cgi?ID=C3010024 | None |
| Ethanol, 2-nitro- | https://webbook.nist.gov/cgi/cbook.cgi?ID=C625489 | None |
