# Supplementary material for: High accuracy machine learning identification of fentanyl-relevant molecular compound classification via constituent functional group analysis
Source: Sci Rep. 2020 Aug 11;10:13569. doi: 10.1038/s41598-020-70471-7 (PMC7419312; doi:10.1038/s41598-020-70471-7)
Supplement: Supplementary file 2 — Supplementary Appendix B. [file 41598_2020_70471_MOESM2_ESM.docx]

**Supplementary Information- Appendix B**

**Title: High Accuracy Machine Learning Identification of Fentanyl-Relevant Molecular Compound Classification *via* Constituent Functional Group Analysis**

**Authors:**  Mengyu Xu,^1,4^ Chun-Hung Wang,^2^ Anthony C. Terracciano,^3,4^ Artem E. Masunov,^2,5-8^ and Subith S. Vasu^3,4, *^

**Affiliations:**

^1^Statistics and Data Science, University of Central Florida, 4000 Central Florida Blvd, Orlando Fl, 32816, USA

^2^NanoScience Technology Center, University of Central Florida, 12424 Research Parkway, Orlando Fl, 32826, USA

^3^Mechanical and Aerospace Engineering, University of Central Florida, 4000 Central Florida Blvd, Orlando Fl, 32816, USA

^4^Center for Advanced Turbomachinery and Energy Research, University of Central Florida, 4000 Central Florida Blvd, Orlando Fl, 32816, USA

^5^School of Modeling, Simulation, and Training, University of Central Florida, 3100 Technology Parkway, Orlando, FL 32816, USA

^6^Department of Chemistry, University of Central Florida, 4111 Libra Dr., Orlando, FL 32816, USA

^7^South Ural State University, Lenin pr. 76, Chelyabinsk 454080, Russia

^8^National Research Nuclear University MEPhI, Kashirskoye shosse 31, Moscow, 115409, Russia

*Corresponding: subith@ucf.edu

**Appendix B Comparison with Other Classifiers**

In this appendix we provide a performance comparison between commonly used classifiers. The IR spectral absorbance strength at each of the 876 wavenumbers is treated as predictor. We perform the high-dimensional classification with the following algorithms: L1-regularized multinomial logistic regression (i.e., logistic Lasso), referred to as MLR; Partial least squares, referred to as PLS; and J48 decision tree classifier, referred to as J48. In the tables and figures below, our result is referred to as fPCA_GLM. For MLR, the tuning parameter $\lambda$ is selected such that at most 30 predictors have non-zero coefficients, as we assume the true predictors are sparse; for PLS, the number of components is selected such that 80% of variance in the predictors is explained. The results are obtained with R packages caret^1^, RWeka^2^ and glmnet^3^ for PLS, J48 and MLR respectively.

In Table B.1 we report the AUCs of the binary classifications for appearance of each functional group in the test set. In Figure B.1, the ROC curves are presented. Table B.2 shows the overall classification accuracy of each classifier.

Except the decision-tree-based algorithm J48, the performance of the other classifiers are similar to each other.

Table B.1 AUCs of the binary classifications for appearance of each functional group in the test set

|  | fPCA_GLM | PLS | J48 | MLR |
| --- | --- | --- | --- | --- |
| Amide | 0.9815 | 0.9907 | 0.8511 | 0.9799 |
| Aniline | 0.9336 | 0.9254 | 0.8693 | 0.9578 |
| Benzene | 0.9019 | 0.9049 | 0.8021 | 0.94 |
| Piperidine | 0.9712 | 0.9148 | 0.7053 | 0.9868 |

Table B.2 Overall accuracy of the multi-label classifications

|  | fPCA_GLM | PLS | J48 | MLR |
| --- | --- | --- | --- | --- |
| Accuracy | 0.7937 | 0.7937 | 0.7460 | 0.8254 |

Figure B.1 Overall accuracy of the multi-label classifications

| 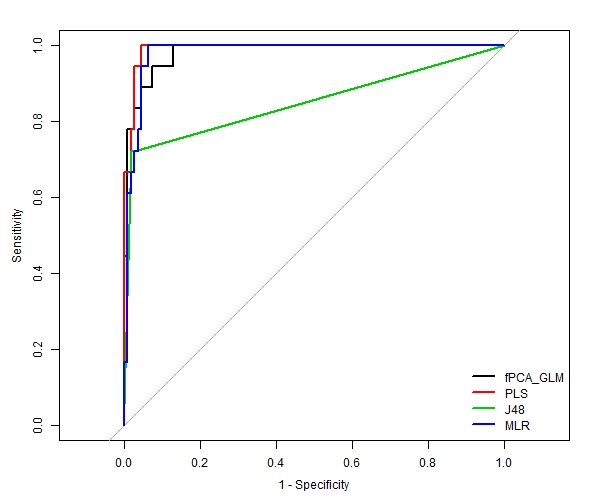  (a) | 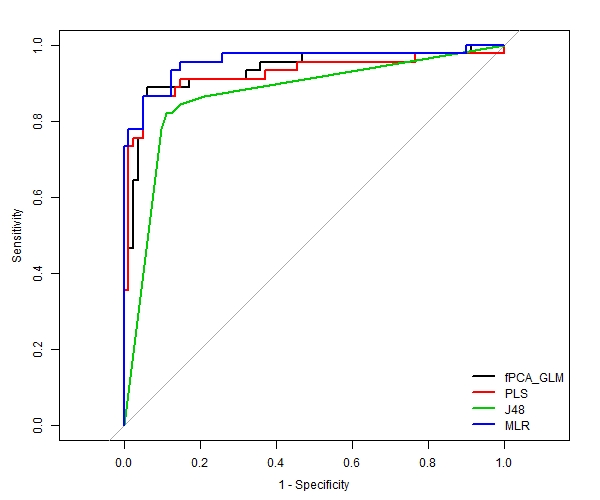  (b) |
| --- | --- |
| 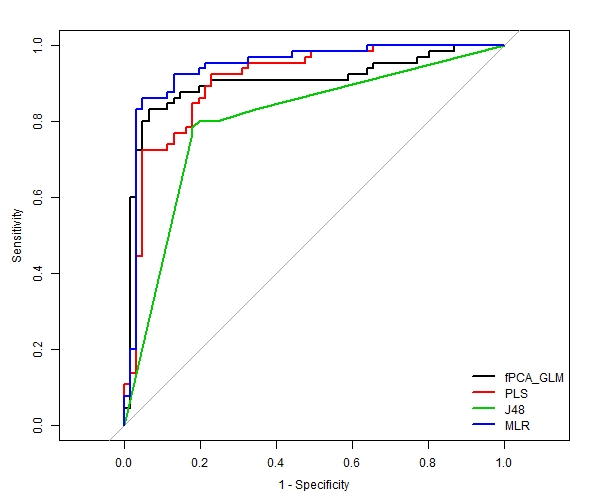(c) | 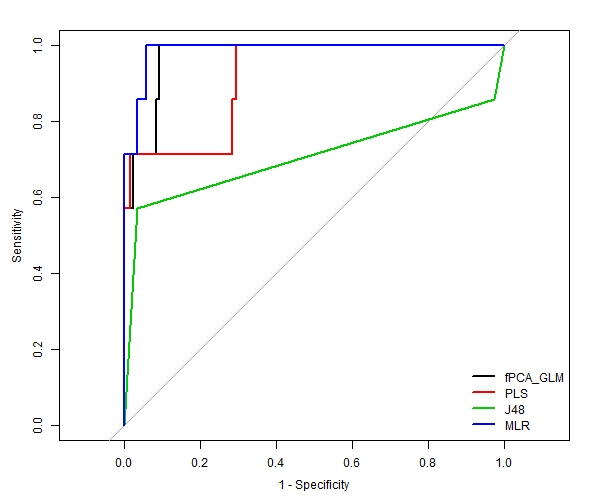  (d) |

1 Kuhn, M. Caret: classification and regression training. *Astrophysics Source Code Library* (2015).

2 Witten, I. H. & Frank, E. Data mining: practical machine learning tools and techniques with Java implementations. *Acm Sigmod Record* **31**, 76-77 (2002).

3 Friedman, J., Hastie, T. & Tibshirani, R. Regularization paths for generalized linear models via coordinate descent. *Journal of statistical software* **33**, 1 (2010).
